# Consensus Statement on Brillouin Light Scattering Microscopy of Biological Materials^§^

**Published:** 2024-11-18

**Authors:** Pierre Bouvet, Carlo Bevilacqua, Yogeshwari Ambekar, Giuseppe Antonacci, Joshua Au, Silvia Caponi, Sophie Chagnon-Lessard, Juergen Czarske, Thomas Dehoux, Daniele Fioretto, Yujian Fu, Jochen Guck, Thorsten Hamann, Dag Heinemann, Torsten Jähnke, Hubert Jean-Ruel, Irina Kabakova, Kristie Koski, Nektarios Koukourakis, David Krause, Salvatore La Cavera, Timm Landes, Jinhao Li, Jeremie Margueritat, Maurizio Mattarelli, Michael Monaghan, Darryl R. Overby, Fernando Perez-Cota, Emanuele Pontecorvo, Robert Prevedel, Giancarlo Ruocco, John Sandercock, Giuliano Scarcelli, Filippo Scarponi, Claudia Testi, Peter Török, Lucie Vovard, Wolfgang Weninger, Vladislav Yakovlev, Seok-Hyun Yun, Jitao Zhang, Francesca Palombo, Alberto Bilenca, Kareem Elsayad

**Affiliations:** 1 Center for Anatomy and Cell Biology, Medical University of Vienna, Austria; 2 Cell Biology and Biophysics Unit, European Molecular Biology Laboratory, Germany; 3 Fischell Department of Bioengineering, University of Maryland, USA; 4 Specto Srl., Milan, Italy; 5 CNR - Istituto Officina dei Materiali (IOM), Unità di Perugia, Italy; 6 LightMachinery Inc., Canada; 7 Departement of Electronics, Carleton University, Canada; 8 Laboratory of Measurement and Sensor System Technique (MST), TU Dresden, Germany; 9 Cluster of Excellence Physics of Life, TU Dresden, Germany; 10 Competence Center for Biomedical Computational Laser Systems, TU Dresden, Germany; 11 Institut Lumière Matière, UMR5306 Université Lyon 1-CNRS, Université de Lyon, France; 12 Dipartimento di Fisica e Geologia, Università di Perugia, Italy; 13 Max Planck Institute for the Science of Light, Erlangen, Germany; 14 Department of Biology, Norwegian University of Science and Technology, Trondheim, Norway; 15 Hannover Centre for Optical Technologies, Leibniz University Hannover, Germany; 16 CellSense Technologies GmbH, Berlin, Germany; 17 School of Mathematical and Physical Sciences, University of Technology Sydney, Australia; 18 Department of Chemistry, University of California Davis, USA; 19 Optics & Photonics Group, Faculty of Engineering, University of Nottingham, United Kingdom; 20 Discipline of Mechanical, Manufacturing & Biomedical Engineering, Trinity College Dublin, Ireland; 21 Department of Bioengineering, Imperial College London, United Kingdom; 22 CREST Optics S.p.A., Rome, Italy; 23 German Center for Lung Research (DZL), Heidelberg, Germany; 24 Center for Life Nano- & Neuro-Science, Istituto Italiano di Tecnologia, Rome, Italy; 25 The Table Stable Ltd., Mettmenstetten, Switzerland; 26 School of Physical & Mathematical Sciences, Nanyang Technological University, Singapore; 27 Lee Kong Chian School of Medicine, Singapore Centre of Environmental Life Sciences Engineering (SCELSE), Nanyang Technological University, Singapore; 28 Institute for Digital Molecular Analytics & Sciences, Nanyang Technological University, Singapore; 29 Department of Biomedical Engineering, Texas A&M University, USA; 30 Department of Electrical and Computer Engineering, Texas A&M University, USA; 31 Department of Physics and Astronomy, Texas A&M University, USA; 32 Harvard Medical School and Massachusetts General Hospital, USA; 33 Department of Biomedical Engineering, Wayne State University, USA; 34 Department of Physics and Astronomy, University of Exeter, Exeter, United Kingdom; 35 Biomedical Engineering Department, Ben-Gurion University of the Negev, Israel

## Abstract

Brillouin Light Scattering (BLS) spectroscopy is a non-invasive, non-contact, label-free optical technique that can provide information on the mechanical properties of a material on the sub-micron scale. Over the last decade it has seen increased applications in the life sciences, driven by the observed significance of mechanical properties in biological processes, the realization of more sensitive BLS spectrometers and its extension to an imaging modality. As with other spectroscopic techniques, BLS measurements not only detect signals characteristic of the investigated sample, but also of the experimental apparatus, and can be significantly affected by measurement conditions. The aim of this consensus statement is to improve the comparability of BLS studies by providing reporting recommendations for the measured parameters and detailing common artifacts. Given that most BLS studies of biological matter are still at proof-of-concept stages and use different--often self-built--spectrometers, a consensus statement is particularly timely to assure unified advancement.

In the context of studying bio-relevant matter, BLS spectroscopy involves measuring the frequency and lifetime of MHz-GHz frequency acoustic phonons (propagating density fluctuations) in a material. This is possible by making use of the constructive and destructive interference of light scattered from propagating thermal or stimulated phonons of a given wavevector determined by the Bragg condition^1^. A BLS spectrum of a homogeneous and isotropic material will consist of at least two (Stokes and anti-Stokes) peaks that reveal the amplitude, frequency and lifetime of acoustic phonons at the probed scattering wavevector(s).

Examples of the BLS spectra of distilled water, measured using different spectrometer designs, are shown in Figures 1A–E. Also shown in each case is the spectrum of (>99% pure) cyclohexane, which has a smaller BLS frequency shift ($v_{B}$) and broader linewidth ($\Gamma_{B}$) than water. A description of the different spectrometer designs can be found in the Supplementary Text. In each case, the frequency shift of the BLS peak corresponds to the probed phonon frequency. Using the wave equation this can be used to calculate the phonon hypersonic speed, which is related to the respective elastic modulus (see Supplementary Text). The width of the BLS peaks is related to the imaginary (dissipative) part of the modulus which can be expressed in terms of the longitudinal viscosity, although it is also dependent on extrinsic experimental conditions and is in general affected by spatial heterogeneities in the sample in a non-trivial manner.

While BLS spectroscopy has been performed in research laboratories for more than half a century^2^, the recent surge of interest for biomedical applications can largely be attributed to its extension to an imaging modality^3,4^ suitable for studying biological samples^4^. Driven also by our growing awareness of the significance of mechanical properties for biological processes^5,6^, the last decades has seen the development of a number of different approaches^7,8^ to obtain BLS spectra of biological matter (both in the frequency and in the time domain, and both from inherent thermal and stimulated acoustic phonons), that have been applied for studying diverse biological and biorelevant systems^8–10^. These, in principle, are expected to yield comparable results for the viscoelastic moduli. Each approach however has distinct experimental challenges, limitations and characteristic sources of uncertainty. There are ongoing discussions on the biological significance of BLS-derived parameters in the context of soft tissue and cells^11–15^, here though we focus on the optics and photonics aspects necessary to extract reliable and repeatable parameters in biological matter. We also only consider BLS performed at visible to NIR wavelengths, as X-ray^16^ and neutron^17^ Brillouin scattering are associated with distinct challenges.

## Parameters that BLS measures

Two *key parameters* measured in BLS spectroscopy are the BLS frequency peak shift ($v_{B}$) and the BLS linewidth ($\Gamma_{B}$), both conventionally presented in units of MHz or GHz. To obtain these parameters, one typically fits each Brillouin peak in the frequency domain with a suitable function (Supplementary Text).

Experimental data for a BLS measurement typically consists of the intensity at different frequencies that spans one or both Stokes and anti-Stokes BLS peaks, or time-series data longer than $1/\Gamma_{B}$ with a sampling frequency $>2v_{B}$. In most cases, BLS measurements do not measure the elastic scattering peak, which is often suppressed, masked or otherwise avoided, due to its large intensity. Below we list some physical quantities that can be extracted from the key parameters (equations for calculating these and others are provided in the Supplementary Text).

### Hypersonic acoustic speed (V):

The phase velocity of the probed acoustic modes in the direction of the scattering wavevector. It is obtained from $v_{B}$, and depending upon the scattering geometry, the refractive index (*n*) of the sample in the direction of the scattering wavevector.

### Longitudinal Storage Modulus ($M^{\prime }$):

A measure of the elastic properties subject to longitudinal boundary conditions. For isotropic materials it is the combination of shear ($G^{\prime }$) and bulk ($K^{\prime }$) moduli: $M^{\prime }\;=\;K^{\prime }\;+\;(4/3)G^{\prime }$ (Supplementary Text). In addition to all parameters required for calculating V, it also requires knowledge of the mass density (⍴).

### Longitudinal Loss Modulus ($M^{\prime \prime }$) and Longitudinal Viscosity ($\eta_{L}$):

A measure of the viscous (dissipative) properties subject to longitudinal boundary conditions. In addition to all parameters required for calculating $M^{\prime }$, it also requires $\Gamma_{B}$.

### Longitudinal Loss Tangent (tanδ):

Defined as $M^{\prime \prime }/M^{\prime }$, which is equal to $\Gamma_{B}/v_{B}$, a measure of attenuation independent of ⍴, and usually to a good approximation n.

### Shear Modulus (G):

Can be obtained from the BLS frequency shift of the Transverse Acoustic (TA) modes in a symmetry direction. TA phonon modes are manifested as two (Stokes and anti-Stokes) distinct peaks to those of Longitudinal Acoustic (LA) phonons, usually with a smaller BLS frequency shift. They typically require measurements at different scattering geometries and polarizations^18–21^. In soft-matter and liquids the frequency shift of the TA phonons may be practically too small to measure. Calculation of G also requires ⍴ and n in the direction of the scattering wave-vector. G is a complex quantity, with the real and imaginary part obtainable from the BLS frequency shift and linewidth in an analogous manner to $M^{\prime }$ and $M^{\prime \prime }$.

### Tensile or Young’s moduli (E), Bulk Moduli (K), and Poisson’s ratios (σ).

These can be derived from measurements of *G* and *M* and require *a priori* knowledge or assumptions on the symmetry of the sample^18–20^. This is usually practical only in hard matter where the frequency of the TA phonon peak is not too small.

### Landau-Placzek Ratio:

Is defined as the ratio of the integrated Rayleigh scattering peak intensity (component of elastic scattering from the thermal diffusivity mode^22^) when measured, to the integrated BLS peak intensity (defined as the total area underneath the respective peak)^1^. For simple homogeneous samples it is equal to the difference between the specific heat at constant pressure and that at constant volume, relative to that at constant volume^1^. This can give insight into the thermodynamic properties of a sample, albeit the validity breaks down in complex materials and an accurate measure of the Rayleigh signal can be challenging in the presence of other elastic scattering processes (e.g. Tyndall scattering^23^). As such, its consideration in complex biological matter has thus far been avoided.

### Refractive Index (n):

Can be calculated for isotropic materials or materials with known symmetry from angle-resolved measurements or from several different scattering geometries^19,24^, under the assumption of a homogeneous sample, and at frequencies not in the vicinity of a structural relaxation process or phase change^25^.

### Mass Density (ρ):

Can be calculated in stimulated BLS measurements via the BLS gain, with which it scales linearly^26^.

As can be seen, calculation of viscoelastic parameters typically require knowledge of extrinsic parameters (⍴ and n), which may not always be accessible. In homogeneous materials, n can be measured using an Abbe refractometer, whereas in thin heterogeneous samples it can be spatially mapped using phase-resolved methods (quantitative phase imaging^27^ or digital holographic tomography^28^). ⍴ can be estimated from n via the Lorentz-Lorenz relation and knowledge of the proportionality coefficient(s)^29^ (Supplementary Text). This however may not be trivial in heterogeneous samples, where these vary spatially. ⍴ may alternatively be obtained using the linear gradient method^30^ and for homogeneous samples using a densiometer^31^ or measurement of sample mass and volume. If ρ and n are not accessible, then an approximation may be made from literature values. For samples with a spatially uniform chemical composition and material phase, a near constant ratio of $\rho /n^{2}$ may be assumed^32^, however this can break down when samples have gradients in composition (e.g. interfaces between hydrophilic-hydrophobic regions).

## Resolution

While the spectral resolution of the spectrometer is a limiting factor, spectral deconvolution^33^ and spectral fitting^34^ can be employed for highly precise measurements of BLS frequency shifts and linewidths (exceeding the accuracy with which $v_{B}$ can be extracted by two orders of magnitude compared to the spectral resolution of the spectrometer^35^). For an unbiased comparison of the spectral precision of different BLS spectrometer setups the Allan variance method, introduced for IR spectroscopy, has been suggested^36,37^. This can be applied to BLS experiments by measuring standard deviations of the fitting parameters over the sampling time^38,39^. In order to obtain unbiased comparisons though, one should bear in mind that spectrometers may allow additional optimization by modulating the mirror spacings, as well as the acquisition/scan times, amplitudes, and number of acquisition points.

The spatial resolution is fundamentally limited by the effective Point Spread Function (PSF) of the microscope itself (with the exception of the axial resolution in Time Resolved Brillouin Scattering (TRBS)–see Supplementary Text). Given BLS is a coherent phenomenon, the effective PSF may be larger than the PSF defined using incoherent probes (e.g. fluorescent labels) or scattering processes. This can be understood in that the probed photon-phonon interactions are dependent on the coherence lengths of the phonons (i.e. the characteristic size over which the density fluctuations are correlated)^40,41^. This is sample dependent and given by ${\Lambda v}_{B}{/\Gamma }_{B}$, where Λ is the phonon wavelength^42^. Increasing the mechanical contrast between different regions typically reduces the phonon coherence and, therefore, improves the spatial resolution up to the limit of the optical resolution obtainable from incoherent scattering processes. In practice, the true spatial resolution is best determined experimentally on mock-up systems, consisting of different materials with sharp interfaces and spatially scanning between these.

## Reporting Consensus

Below we describe parameters we consider important to report in all BLS measurements, regardless of the spectrometer used. These are provided also in the form of a *Minimal Reporting Table* (available at: DOI:10.6084/m9.figshare.27794910)

1. ***Spectrometer*.** In addition to information on the type of spectrometer, this should also include its Free Spectral Range (FSR), sampling step size and range (in frequency for frequency domain or time for time domain).
2. ***Spectral resolution.*** In frequency-domain spectrometers this is often most easily obtained from measuring the elastic scattering peak width from a highly scattering sample (e.g. mirror flat), with the assumption that the probing laser(s) have much narrower spectral line widths than the frequency shifts measured. In time-domain measurements this can be estimated from the repetition rate of the pulsed laser, which acts as the frequency comb spacing in the spectral domain. The spectral resolution is generally reported in units of MHz. While the spectral resolution is not a direct indication of the precision with which the key parameters can be extracted if the shape of the peak is known, it is important for distinguishing multiple peaks (which may occur near interfaces^43^) or deducing undefined/uncertain functional peak shapes (which may occur near structural transitions).
3. ***Spectral precision.*** This is distinct to the spectral resolution in that it is a measure of how precisely $v_{B}$ and $\Gamma_{B}$ can be determined from repeated measurements, and ultimately a measure of the stability of the microscope/spectrometer. A reasonable practical measure of this can be obtained from the spread of repeated measurements or a spatial scan of a static homogeneous sample. In certain cases the difference in the absolute frequency shift and linewidth of the Stokes and anti-Stokes peaks may also contribute and should be considered. These parameters are generally reported as coefficients of variation on the mean (ratio of the standard deviation on the mean by the mean) as percentages (%). For a heterogeneous or time-varying sample the precision can have inherent spatio-temporal variability that is not accounted for with a single spectral precision, which should be considered. The acceptable spectral precision will depend on the size of the changes in the key parameters one wishes to discern. In practice a ~0.5% precision for $v_{B}$ and $\Gamma_{B}$ often suffices for discerning significant changes in the derived elastic and viscous parameters of biological samples.
4. ***Signal to Noise Ratio (SNR) and Signal to Background Ratio (SBR).*** These are measures of how much the BLS signal compares to all other non-BLS detected signals. Various sources of background signals can exist including residual Amplified Spontaneous Emission (ASE), side modes of laser sources that are not perfectly clean, fluorescence signals, and artefacts from elastic scattering (e.g. from turbid samples like tissue or bones). The most desirable operating conditions are that all non-BLS signals are removed and that enough BLS photons are collected to operate in the shot noise limit, considering also the detector used. Reporting the SNR and SBR is therefore important to demonstrate if the measurement is shot noise (highly preferred) or background limited. Here the SNR may be quantified in an analogous manner to what has been used in Raman spectroscopy^44^, as the ratio of the averaged BLS peak intensity to its standard deviation, while the SBR is the ratio of the averaged Brillouin peak intensity to the standard deviation of the noise floor in the absence of the Brillouin signal.
5. ***Numerical aperture (NA) of excitation and detection light / Scattering angle(s).*** Since $v_{B}$ and $\Gamma_{B}$ are dependent on the scattering angle, using high NA’s (>0.4) will result in broadening of the linewidth due to the increased spread of scattering wavevectors measured^45,46^. At scattering angles away from backscattering the effect of finite-NA becomes particularly pronounced and an experiment specific trade-off needs to be made on acceptable spread in scattering wave-vector, spatial resolution, laser exposure and acquisition time. While conventional wisdom states that increasing the excitation and/or detection NA results in a higher lateral spatial resolution, in BLS microscopy this may not always be the case on account of the finite phonon length scales^42^. As such, explicitly reporting both the excitation and detection NA or angles is essential for meaningful interpretation of data. In addition, for confocal implementations, reporting of the effective size of the detection pinhole (physical pinhole or fibre core diameter), *e.g.* in Airy Units, is important for understanding the confocality as well as the probed wavevectors.
6. ***Average Power/Peak Power at Sample/Pulse repetition rate.*** Measurements conducted at low average laser power have the advantage of reduced photodamage/phototoxicity for live cell and tissue studies. They also reduce the potential of significant sample heating which affects the BLS spectrum. While for biological samples it is almost always desirable to use as low of a laser power as possible while still having an acceptable SNR, for dynamic/moving samples there may be a trade-off to capture the desired dynamics. In regard to not perturbing sensitive biological processes the total laser exposure (energy deposited on sample) is often more relevant, and as such a balance between exposure time and laser power may need to be found. The acceptable power-intensity is ultimately sample dependent and guidelines set out for other microscopy techniques should be followed^47^.
7. ***Laser wavelength(s).*** $v_{B}$ and $\Gamma_{B}$ depend on the wavelength of the laser source. When selecting wavelengths, both the strength of the BLS signal, the potential for photodamage as well as how deep inside a sample one wishes to measure, should be considered. While the BLS scattering cross-section scales as $\lambda^{-4}$ and the spatial resolution of a measurement increases with decreasing λ, the photodamage to biological materials is generally more significant at shorter wavelengths.
8. ***Exposure/Acquisition Time*.** While the exposure time per spectral acquisition should be chosen to obtain sufficient SNR, dynamic/moving samples may set a practical upper limit, in which case a compromise of increasing laser intensity and decreasing spectral resolution may be needed.
9. ***Sample Temperature*.** Given the strong temperature dependence of BLS, we recommend sample temperature should be reported to within +/−0.5°C accuracy. This will typically cause errors in the value of $v_{B}$ in hydrated samples of less than ≈0.1%.

## Methods

A description of the different approaches for performing BLS spectroscopy, together with measurement and reporting recommendations particular to each, are presented in the Supplementary Text. Here we discuss aspects relevant to different spectrometer designs, as well as the comparison of measurements on different setups.

### Calibration spectra

BLS spectra need accurate and robust calibration for data to be comparable across labs, experimental conditions, and over time. For instruments needing calibration using a reference inelastic peak, such as Virtually Imaged Phased Array (VIPA) architectures or single etalon systems, optimal performance is achieved by eliminating the elastic scattering, and calibration is typically performed using materials with known $v_{B}$ to estimate the unknown dispersion parameters. The accuracy of these calibration procedures rely on the accuracy of the reference $v_{B}$, which may depend on other experimental parameters such as temperature (e.g. $v_{B}$ of water increases by ~1%/°C; whereas $v_{B}$ of polystyrene decreases with increasing temperature^48^).

Calibration based on the absolute, and highly conserved, absorption lines of atomic gas vapours such as rubidium may be employed. It has been shown that locking the laser frequency to such narrow absorption lines allows for calibrated measurements that are accurate to within a few MHz over extended periods^49^. Alternatively, a robust calibration strategy can involve spectrally shifting a part of the elastic scattered signal by a few GHz using an electro-optic modulator (EOM)^50–52^ or potentially also an acousto-optic modulator (AOM).

### Fitting of BLS spectra

In cases where the presence of a mechanical relaxation process can be ruled out, the BLS peak is well represented by a Damped Harmonic Oscillator (DHO). When spectra are measured using Fabry-Perot interferometers or monochromators, a direct DHO fit is feasible. However, for imaging spectrometers where a spectral projection is generated by introducing a small divergence to the light incident on an interferometric element, and the signal is dispersed into one or more orders, the spectral projection will be weighted by a Gaussian envelope^53^. This results in a slight functional correction, and fits are typically performed using Lorentzian functions. Since the parameter $v_{B}$ in a DHO fit does not exactly coincide with the peak maximum a, usually small, correction may need to be applied for broad BLS-peaks when using Lorentzian fitting^54^.

In the ideal scenario, the detected signal is shot noise limited, such that the variance σ scales as $\sigma_{i}^{2}=N_{i}$, where $N_{i}$ is the number of photons detected in a given frequency interval. The large variation in the relative photon count uncertainty across the BLS peak thus requires a weighted ($\propto \;\sigma_{i}^{-2}$) regression routine for fitting. In multi-pixel detectors there may also be an additional source of noise, e.g. due to electron amplification, that increases the variance, which should be considered. The fitting function may thus also include a baseline term to account for this and other physical phenomena (e.g. fluorescence and Raman scattering *folded* into the spectral region of interest due to the finite FSR of the spectrometer). This contribution and its uncertainty should be considered in the computation of the SNR. Rather than subtracting such contributions from the spectra, which can be statistically misleading, they should explicitly be included in the fitting function.

The measured BLS spectrum is always subject to instrumental broadening and should thus ideally be deconvolved with the (spectral) Instrument Response Function (IRF). Alternatively, a modified fitting function can be constructed by convolving the model function (DHO or Lorentzian) with the IRF. In frequency domain spectroscopy the IRF can be obtained by measuring the spectra of the elastically scattered probing laser through the optical setup by replacing the sample with a mirror flat in the backscattering geometry.

### Uncertainties

When reporting the uncertainties of a BLS measurement, it is recommended to follow the guidelines established by the international organisations of metrology^55^. Whenever possible, repeated measurements of the sample under the same conditions should be conducted to evaluate the uncertainty caused by random error sources. This type of uncertainty (Type A evaluation) is expressed as the standard deviation of the mean. For uncertainties that cannot be estimated from repeated measurements (Type B evaluation), such as systematic errors, a comparison with the reported values of standard materials should be performed when possible. The combined uncertainty can then be calculated by considering the dependencies between error sources. To mitigate systematic errors, regular calibration with readily available homogeneous materials with constant $v_{B}$ values, and where the BLS peak is well described by a model fitting function, is recommended. To report the uncertainty of any calculated viscoelastic parameters, the uncertainties of each of the measured or assumed parameters should be evaluated separately and then combined according to the law of propagation of uncertainty.

### Parameters for high-quality BLS measurements

One can distinguish between parameters that concern the illumination (probing) light and the detection of the BLS signal. On the illumination side, the most important parameters are: (1) *laser linewidth*. This determines the upper limit of the achievable spectral resolution, and should typically be <100 MHz. (2) *Long term laser wavelength stability*. This should ideally be <1GHz drift per hour. Locking the laser wavelength to a gas absorption cell line or re-calibration with control samples may be employed. (3) *Spectral noise of the laser* (amplified spontaneous emission--ASE and “side modes”) can lead to considerable spectral contributions either side of the main laser line. ASE influences the spectral noise floor from its elastic scattering by the sample or within the optical setup. It can, on account of its spectral breadth, be challenging to filter out, potentially even requiring a combination of etalon filters. Typically, the total power outside of the main laser line should be ~60–90dB lower than that of the main laser line for high-quality BLS measurements.

On the detection side, it is most important to sufficiently suppress elastic scattered light. The required magnitude thereof depends on a sample’s scattering properties, although a suppression of ~60–90dB (less in homogenous, low-scattering samples such as transparent liquids, more in highly scattering samples such as calcified bones) is desirable. At the same time, the detection of the BLS signal needs a sufficiently high SNR for high-precision fitting. If a (typical) precision of ~10MHz is desired, this requires an SNR of between ~30–50, depending slightly on other spectrometer properties and acquisition parameters (spectral sampling, etc.)^56^. Given that state-of-the-art spectrometers are often shot noise limited, this would correspond to a total signal that is on the order of 10^3^ – 10^4^ detected photons across the measured BLS spectrum.

While a high SNR, spectral resolution and precision will result in more reliable determination of the key parameters, how accurately they can be extracted ultimately depends on how accurately the fitting model describes the measured spectra. For this including finite-NA peak broadening, multiple scattering in highly scattering samples, background noise, and deconvolving with the IRF will all play a part.

### Artifacts

Different spectrometer designs will have unique sources of artifacts, which are described in the Supplementary Text. Below we list artefacts that were identified as being common to several spectrometer designs, and which can affect determination of the BLS key parameters.

#### Material (acoustic) heterogeneities:

When elastic heterogeneities exist in the scattering volume, the BLS spectrum may no longer exhibit a single set of peaks (per acoustic mode). While not necessarily an artefact, since it can provide insight into the composition of a sample, it nevertheless may require explicit consideration. The effect will depend on the size and the acoustic mismatch of the heterogeneities^40,41,57^. When the size of the acoustic heterogeneities is larger than the phonon coherence length, one will have distinct peaks associated with the different materials that can be analyzed independently^58^ (with an intensity proportional to the product of the filling factor and scattering efficiency^57^). If the size of the heterogeneities is less than the phonon coherence length and there is a significant acoustic mismatch associated with the heterogeneities, the acoustic modes will be confined, and the BLS spectra will exhibit peaks whose position is a function of their morphological and acoustic properties. Here a specific analysis procedure is required^41,59,60^. Finally, if the phonon coherence length is larger than the heterogeneities, one may assume an effective material response. Here the heterogeneities cause a *static* attenuation process, which adds to the inherent dynamic ones, and is associated with an increase in $\Gamma_{B}$^40^.

#### Scattering wavevector indeterminacy:

An indeterminacy in the probed scattering wavevector (*q*) will be reflected in both $v_{B}$ and $\Gamma_{B}$, and any subsequently derived parameters. Given that the probing and collection optics have a finite NA, this effect is unavoidable. In the case of low NA and in the back scattering configuration this effect is largely reduced^46^. However for high NA excitation or detection, as well as for small scattering angles^61^, the effect on both $v_{B}$ and $\Gamma_{B}$ can become very significant^42,45^. Given that the theoretical evaluation of the *q*-indeterminacy may not be straightforward, it can be desirable to estimate this from measurements on materials with negligible intrinsic broadening, which can serve to also obtain the IRF of the optical setup.

In turbid media where one has significant multiple scattering, the effects of *q*-indeterminacy becomes more complicated, as the probed phonons are no longer defined by the external scattering geometry. Here the observed spectrum is a complex superposition of BLS spectra with different scattering geometries. Methods based on e.g. polarization gating^62^ may be used to separate different contributions and extract the true BLS parameters.

#### Sample heating effects:

Temperature has a significant effect on the BLS key parameters as well as cellular processes. In hydrated biological samples an increase in temperature will typically result in an increase of $v_{B}$ and decrease of $\Gamma_{B}$. As such it is important to consider uncertainties in the key parameters due to uncertainties in the temperature. To check whether local sample heating is significant, it can suffice to perform equivalent measurements with different laser intensities or sample exposure times.

Given that some BLS approaches require intense and/or extended sample laser exposures, sample heating may become relevant. In general, this is non-trivial to calculate or measure in-situ and will depend upon numerous parameters. Semi-quantitative estimates can be made using *e.g.* the bio-heat equation^63^.

In time-domain BLS the temperature rise at the transducer caused by the absorption of laser pulses may also contribute to sample heating. This is most significant when using large average laser powers and thermally insulating substrates. Using a sapphire substrate with 1.5 mW average laser power, it was shown that one would have a ~1°C temperature rise in the sample measurement volume^64^.

#### Laser frequency drifting or line-broadening:

Ideal lasers for Brillouin scattering are narrow linewidth (< 10MHz) at a single, stable frequency. Some commercial continuous wave (CW) single-longitudinal mode lasers, such as those commonly used in spontaneous and stimulated frequency-domain BLS, experience frequency drifts, or line-broadening, that are predominantly caused by temperature fluctuations. Such frequency drifts can be >100 MHz for environmental temperature variations of 0.1°C, although the amount will vary depending on the laser type and manufacturer^65^. To increase wavelength stability during BLS spectra acquisitions, laser sources may be actively locked to a reference (*e.g.* Rb/I_2_ gas cell, or a stable external cavity). Depending on the time scales associated with frequency drifts, one may perform periodic measurements of a reference material or signal with known $v_{B}$ and implement frequency drift corrections during analysis.

#### Elastic scattering filters:

Given the tight frequency-matching requirements, stability represents the main challenge for most elastic scattering filters. The suppression of the elastically scattered light can be compromised by laser frequency drifts, mechanical drifts, environmental thermal fluctuations, as well as intrinsic Amplified Spontaneous Emission (ASE) from the laser. As such, the use of laser lock-in or closed loop configurations that involve a continuous readout of the elastic scattering signal, are generally required for both interference and molecular absorption-based filters. For the latter, the multitude of absorption lines (especially in I_2_ absorption cells) may attenuate or distort the BLS peaks, which may need to be considered during analysis.

#### Spectral overlaps:

BLS spectrometers operating on interferometric principles will have a finite FSR. In measuring a sample that has a $v_{B}$ larger than the FSR, the BLS peak(s) will appear in higher orders or overlapped orders, if the spectrometer is not explicitly designed to cancel out overlapping and higher order peaks^66^. For example, if a sample has $v_{B}=45\;GHz$ yet the spectrometer FSR is 30 GHz, the BLS anti-Stokes peak will deceptively show up at 15 GHz. This ambiguity, and the measurement of $v_{B}$ values larger than the FSR of a spectrometer, can be overcome by measuring the BLS peaks in higher order ranges^67^. In dual etalon TFP setups such spectral overlaps are eliminated by selecting different etalon FSRs^68^. The issue may also be mitigated in dual-VIPA setups by using VIPA’s with different FSRs effectively increasing the FSR of the spectrometer^69^. This ambiguity in $v_{B}$ will not be an issue in stimulated and time-domain BLS.

#### Internal reflections in sample:

For samples that are thin, highly reflective, or support optical waveguiding modes, multiple reflections from interior surfaces can give rise to additional BLS peaks in non-backscattering geometries^70,71^. This is due to light reflected in the interior of a sample leading to two (or more) inner scattering angles. The result is the observation of two (or more) sets of BLS peaks, one associated with the experimentally chosen scattering geometry and the other corresponding to backscattering from internal reflections. The (longitudinal) modes with the largest $v_{B}$ will generally be those associated with backscattering, and may show angular dependence with sample rotation^70^. Since transverse modes are forbidden in backscattering for high symmetry conditions, only longitudinal modes will appear from such internal scattering and can be identified by employing crossed polarizers in the probing and detection beam paths.

**Figure 2:**
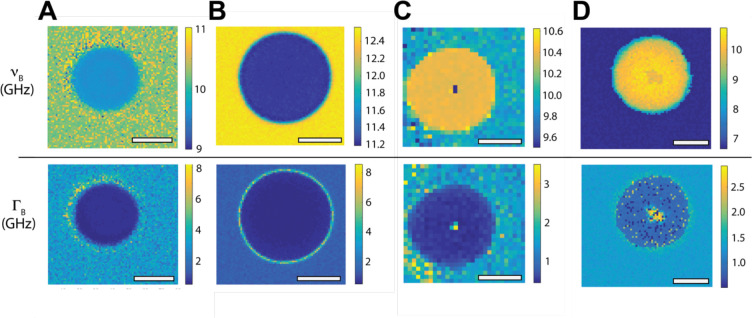
Spatial maps of BLS frequency shift ($v_{B})\;and\;linewidth\;(\Gamma_{B}$) for a 10μm polystyrene bead. **A:** TRBS microscopy measurement with 758nm probing wavelength. **B-D** VIPA-spectrometer measurements with 660nm probing wavelength. A & B were embedded in glycerol (>99%), whereas C and D were measured in glycerol with a significant water fraction and Optiprep^™^ density gradient medium respectively, that both have a lower acoustic index than the bead. Scale bars = 5μm. *Minimum Reporting Tables* for measurements can be found at DOI:10.6084/m9.figshare.27794916.

To illustrate some potential artifacts that can occur in BLS microscopy, in Fig 2A–D we show spatial maps of the BLS frequency shift and linewidth for a 10 μm diameter polystyrene bead measured in four different laboratories. Experimental details can be found in the respective *Minimum Reporting Tables* (available at DOI:10.6084/m9.figshare.27794916). In Fig 2A and 2B the bead has been embedded and sealed in pure (>99%) glycerol which has a larger BLS frequency shift than the bead. In Fig 2C and 2D the embedding medium is glycerol exposed to air for ~24 hours, and an aqueous solution respectively, whose BLS frequency shift is less than that of the bead. These two scenarios (Fig 2A and 2B versus Fig 2C and 2C) create distinct acoustic and optical interfaces between the bead and its surroundings, which can lead to distinct imaging artifacts.

The significant increase in the linewidth at the edge of the bead in Figures 2A and 2B can be partly attributed to the probing volume containing both the bead and the surrounding media (and thus contributions from phonons in both materials), resulting in what is effectively a broader peak. In addition, the acoustic impedance mismatch presented by the curvature of the bead surface may result in an increased spread of scattering angles away from true backscattering. These are hard to avoid given the spherical geometry of the bead, and can also explain the intermediate values of the BLS frequency shift observed at the bead surface in Figure 2B. Abrupt acoustic and optical interfaces generally present a challenge in BLS microscopy, often best addressed by fitting two Stokes and two anti-Stokes BLS peaks in their vicinity.

The region of decreased $v_{B}$ and increased $\Gamma_{B}$ seen at the center of the beads in Figures 2C and 2D, can be understood as a geometrical artifact of the bead acting like a lens, as would occur when the bead is immersed in a lower refractive index media. Measurements at this central position would as a result be over a broader range of also smaller scattering wavevectors (effectively decreasing $v_{B}$ and increasing $\Gamma_{B}$ as seen). The bead represents an extreme example due to the atypically large refractive index and acoustic impedance mismatch with its surroundings (rarely encountered in soft biological matter), but can serve as a useful illustration of potential geometric and material artifacts.

### Comparison of absolute and relative BLS measured values

**Figure 3:**
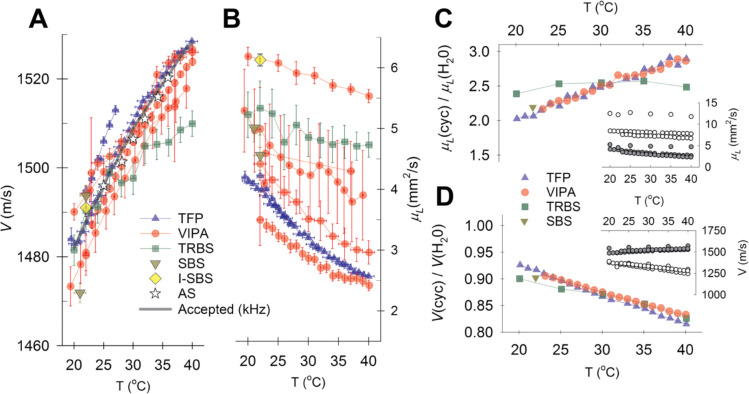
Comparison of Brillouin Light Scattering derived parameters from different spectrometers. **A & B** Hypersonic speed (V) and kinematic longitudinal viscosity ($\mu_{L}$) as a function of temperature measured using different spectrometer designs in participating laboratories. Also shown are accepted values for the acoustic speed in water, and values reported from Acoustic Spectroscopy (AS) measurements^72^. **C & D** BLS obtained $\mu_{L}$ and V of cyclohexane relative to that of pure water measured using different BLS techniques after normalization to Vienna-Hanover water standard--see Supplementary Text. Insets show absolute values for water (filled circles) and cyclohexane (open circles).

Figures 3A and 3B show the acoustic speed ($V=2\pi v_{B}q^{-1}$) and kinematic longitudinal viscosity ($\mu_{L}=2\pi \Gamma_{B}q^{-2}$) of distilled water obtained from measurements on different spectrometers in 15 independent laboratories around the world. Despite measuring at different wavevectors and scattering geometries, all results should yield the same values of V and $\mu_{L}$. Values of the $v_{B}$ derived V are in good agreement between laboratories and spectrometer designs (typically within <0.5%). The $\Gamma_{B}$ derived $\mu_{L}$, while all having similar trends with respect to temperature, show a much larger variability. This is likely due to numerous compounding effects that need to be addressed on a case-per-case basis, including lack of proper spectral deconvolution, finite time windows used to generate spectra (for I-SBS and TRBS), and inadequate corrections for finite probing/detection NA. Among a given spectrometer design the latter presumably gives the largest contribution to the observed variability, since the combined linear and quadratic wavevector dependence of $v_{B}$ and $\Gamma_{B}$ means that even a small change in NA can result in a significant change in the measured $\Gamma_{B}$.

Despite the large variability in the measured $\mu_{L}$, it is possible to obtain comparable absolute values of $\mu_{L}$, provided one corrects for setup specific offsets using e.g. a control sample with known values. Figures 3C and 3D show $\mu_{L}$ and V for >99% pure cyclohexane relative to that of injection grade water measured with a TFP, VIPA, TRBS and SBS spectrometer. In each case a correction factor was applied (see Supplementary Text) based on how the values of $\mu_{L}$ and V for injection grade water differed from those of deconvolved measurements of injection grade water measured using a TFP in Vienna and Hannover and interpolated for all temperatures (made available at DOI:10.6084/m9.figshare.27794913). The differences in the TRBS results may be due to the high acoustic attenuation of cyclohexane compromising the number of discernible oscillations in the time traces, and thereby the Fourier transformed frequency spectrum.

## Conclusions & Discussion

BLS microscopy is an emergent biological imaging technique that provides a unique contrast based on the high frequency acoustic properties of a material. These can be used to calculate the associated viscoelastic parameters and render near optical diffraction limited spatial maps of these. Being label-free and minimally invasive it shares a comparable platform to imaging modalities such as Raman microscopy. Due to its narrow spectral window of excitation and emission, BLS can be performed simultaneously with other imaging techniques (Raman^73^, Fluorescence^74^, etc.) allowing for multimodal imaging.

There are a number of differences between the current consensus statement and consensus statements for other spectroscopic techniques^75^ and techniques that explicitly probe viscoelastic properties. Most notably, many of the instruments addressed here are largely self-built, and differ both conceptually (time-domain, spontaneous, stimulated) and with respect to their unique sources of uncertainty. These complications are contrasted by there usually only being two key parameters over a narrow frequency bandwidth that are of interest. While good agreement can be obtained between laboratories and instrument designs for the BLS frequency shift derived parameters ($V,\;M^{\prime }$), there is significant variability in the reported BLS linewidth derived parameters ($M^{\prime \prime },\eta_{L},\mu_{L}$) which are much more sensitive to experimental configuration. Nevertheless, correcting to a standard sample (such as the Vienna-Hannover measurements on injection grade water done here) can bring these into quantitative agreement amongst different laboratories and spectrometer designs.

Currently the broader implementation of BLS microscopy is, to a large extent, limited by the lack of commercial instruments that can be seamlessly integrated into biological imaging research laboratories, with setups for the most part found in laboratories focusing on optical microscopy development. It however has the potential to fill an important gap in the toolbox of technologies that can quantitatively measure changes in mechanical properties of biological specimens with high spatio-temporal resolution in 3D. BLS can also offers unique possibilities, inaccessible to other techniques that probe mechanical properties, such as quantitatively measuring^7,8^ and mapping^15,18,19,76^ the mechanical anisotropy (different stiffness tensor components), refractive index^24^, as well as combined shear and longitudinal viscoelastic parameters^18–21^.

Already in the life-science research and pre-clinical areas, the value of establishing a consensus is important to reliably compare measurements performed by different instruments, as well as for the commercialization of spectrometers. For the latter it can serve as a blueprint for parameters that need to be documented, and a guide in designing instruments with performance parameters suited for diverse bio-applications. It may also serve as a springboard for formulating more rigorous and application-specific measurement and reporting standards, needed in diverse commercial and medical applications.

To this end we also advocate for the establishment of a common file format to store raw as well as processed BLS spectral data, together with important metadata to understand the context of any given experiment. For this, the popular and well-accepted HDF5 file format^77^ could provide a versatile solution, as it allows one to store complex imagery as well as underlying raw data in a hierarchical and well-structured manner. A first proposed specification for this file format, together with code that allows one to export data to this format, are available in Ref^78^. We foresee to fine tune this format in the future through input from the academic as well as industry community. We expect this standardization in file format to significantly facilitate collaborations, mutual comparisons of experimental results, as well as promote more universal data analysis software development.

While BLS is still in its infancy in regard to clinical translation, one area where it has already made the jump to clinical applications is that of ophthalmology. Here it is being used to identify the severity of pathologies such as keratoconus which are associated with spatial changes in corneal biomechanics^79–81^. As BLS technological sophistication continues to develop in areas such as acquisition speed enhancement^82–85^ and reduction of instrumentation footprint^86^, it simultaneously is also developing in application specific directions (such as endoscopes^87,88^ and versatile fiber-coupled probes^89–92^). One can foresee diverse potential clinical applications that offer improvements over current clinical mechanical sensing (such as palpation and ultrasonic elastography in terms of resolution), as well as novel in-vitro mechano-histopathological^84,93–95^, cellular^96,97^ and biofluid-based diagnostic applications^90,98–100^. Along such clinical translational pathways additional sample-type specific consensus points will no doubt need to be established, as has been the case for more mature technologies such as optical coherence tomography, photoacoustic imaging, Raman and infrared spectroscopy.

As a field, BLS microscopy can be expected to progress in three complementary directions: (1) where BLS-measured viscoelastic parameters provide unique insight into biophysics inaccessible to other techniques that give fundamental insight into material properties and processes in biological systems. (2) Where empirical or theoretically substantiated correlations with viscoelastic parameters measured at different frequencies and boundary conditions can be made, and it serves as a reliable proxy for these. (3) Where BLS measured parameters provide a unique and useful contrast agent on account of their empirical correlation to a biologically relevant material state or process of interest (e.g. a specific pathology). Here they might in of themselves be accepted as presenting a complex chemo-physical property, related to mechanical properties in so far as the measured hypersonic acoustic quantities are. Regardless of the field’s trajectory, it currently finds itself in the somewhat serendipitous position of the technology being ahead of our ability to appreciate the measured parameters’ full biophysical/biological significance. To this end establishing consensus, such as the one presented here, early on is particularly important to assure meaningful and consistent progress on all fronts.

## Supplementary Material

Supplement 1

## Figures and Tables

**Figure 1:**
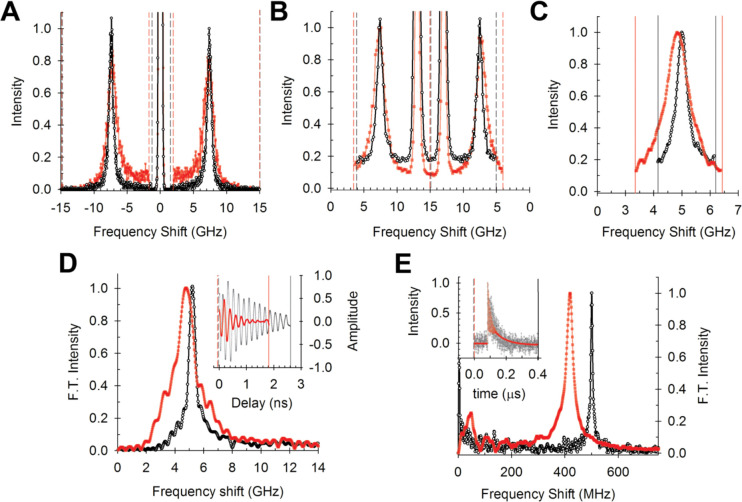
Brillouin Light Scattering Spectra of Liquids. Example BLS spectra of distilled water (black circles) and cyclohexane (red squares) measured using different spectrometer designs (normalized to unity). **A** Tandem Fabry Perot (TFP) spectrometer, **B** Virtual Imaged Phase Array (VIPA) spectrometer with Electro-Optic Modulator (EOM) reference peaks at 13 GHz, **C** Stimulated Brillouin Scattering (SBS) spectroscopy, **D** Time Resolved Brillouin Scattering (TRBS) microscopy and **E** Impulsive SBS (I-SBS) spectroscopy. Insets of D and E show the respective measured time resolved data. The more pronounced difference between water and cyclohexane in E compared to A-D is due to the BLS frequency shift in I-SBS being independent of the refractive index.
