# Supplementary material for: Consensus Statement on Brillouin Light Scattering Microscopy of Biological Materials
Source: ArXiv. 2024 Nov 18:arXiv:2411.11712v1. Preprint. [Version 1] (PMC11601801)
Supplement: Supplement 1 [file NIHPP2411.11712v1-supplement-1.pdf]

Supplementary Text

Consensus Statement on Brillouin Light Scattering Microscopy of Biological Materials

Content:

1: Different BLS instruments & reporting recommendations……..………..…....*p23-p35*

Spontaneous BLS………………………………………………………………...…p23

Single etalon spectrometers…………………………………………………..p23

Multi-pass Fabry-Pérot interferometers……………………….………..p23

VIPA-based spectrometers………...………………………………..………..p24

Crossed VIPAs…………………………………………………………p26

Crossed VIPA-echelle grating………………………………………..p27

Elastic suppression filters …..…………………………………………..p27

Gas absorption cell……..……………………………………..p28

Etalon-based……..…………..………………………………..p28

Radial VIPA………………………………………………………………..p29

Stimulated BLS……………………………………………………………………...…p29

Frequency domain SBS…………………………….………………………p30

Impulsive SBS…………………………………………….…………………p30

Time domain BLS…..……………………………….………………………p30

Heterodyne BLS………………………………………………………..……………...…p33

Fiber probes…..,……………………………………………………..……………...…p34

2: Some useful equations for BLS microscopy…….…….……….……….....…*p36-p49*

Fitting equations……………………………………………………………..…...…p37

Frequency shift……………………………...……………………………..…...…p37

Linewidth…………………………………………………………………...…...…p40

Loss tangent………………………………………………………………...…...…p42

Relation to other moduli.……………………………………………...…...…p42

Calculation of stiffness tensor………………...…………………...…...…p43

Registration to accepted/standard values…………………………...…...…p48

Relation between refractive index and mass density………………...…...…p49

Supplementary References………………………………………………….……*p50-p56*

1: Different BLS instruments & reporting recommendations

Spontaneous BLS

Spontaneous BLS, broadly refers to the case where the probed phonons are not being generated/stimulated by some additional means (e.g. by additional lasers or mechanical transducers). To this end there is typically only a single probing laser, from which the scattered light is analysed, to reveal the scattering from inherent thermal phonons. Currently spontaneous BLS techniques are exclusively confined to the frequency domain, namely they measure the frequency spectrum of the scattered light. In the following subsections, we describe some details and specifics to instruments used in spontaneous BLS measurements.

Single etalon spectrometers

The simplest setup for a Brillouin is a non-scanning single pass, single etalon system. This yields a standard Fabry-Perot set of fringes where the center is the zeroth order and each successive mode is a higher order with non-linear dispersion between orders. Orders are Rayleigh peaks; surrounding each order is the set of Brillouin Stokes and anti-Stokes pairs. In a single etalon system, the light has to be angularly dispersed through the etalon either by tilting or diverging the beam. A cylindrical lens can be used to compress the dispersing light to a line rather than illuminating the entire circular Fabry-Perot pattern. FSR range between orders requires calibration and linearization yet multiple orders can be used for spectroscopic information. Experimental parameters important to report with a single etalon system are the manufacturer, air-gapped (and the gas if different than air) or solid etalon, the flatness, free-spectral range, finesse, how many orders are used in analysis, and whether the spectra is linearized or not linearized.

Multi-pass Fabry-Pérot interferometer

A Tandem Fabry-Pérot spectrometer (TFP) relies on a tandem combination of two air spaced etalons whose spacing is precisely adjusted and controlled. By varying the mirror separation of the etalons, the device sequentially scans consecutive wavelengths. Multiple passes through the etalons are used to increase contrast and finesse. One popular example of this is the 6-pass TFP spectrometer TFP-2 HC, a commercial device developed by Table Stable Ltd., which can achieve a contrast of up to 10^{15}. This high contrast can suppress the elastic background in even strongly turbid samples[73].

The FSR can be varied between about 5 THz and 5 GHz, with a finesse of 100-120 giving a maximum resolution of about 50 MHz. The standard scan time is ~500 ms, but the speed and acquisition bin size can be modulated allowing for e.g. selected spectral windows to accumulate more photons quicker and thus for faster measurements. Using external timing sources, a commercial TFP-2 HC allows faster scanning up to a factor 3-5. A faster version of the TFP, capable of reaching full scan times of 30 ms without any loss of performance, has

been developed successfully by the company Table Stable Ltd. and will be commercialised soon.

A major advantage of the scanning Fabry-Perot is that it is an absolute instrument: the wavelength calibration is determined uniquely by the mirror spacing which is typically known to ~0.1% or better. As such no calibration standard is required, and under laboratory conditions such an instrument will stay stable for hours and even days. Transmission efficiency can be checked by comparison to known samples. When frequency locked to the laser wavelength, there is no theoretical limit on how long such an instrument can be held stable.

When publishing measurements, in addition to the spectrometer manufacturer, one should report the mirror spacing, pinhole sizes (entrance pinhole to spectrometer and exit pinhole to detector), scan amplitude(s), total recording time per channel, Quantum efficiency (QE) of the photon detector used, and a full description of the optics feeding the signal into the TFP.

VIPA-based spectrometers

VIPA (Virtual Imaged Phase Array) spectrometers are non-scanning systems that capture the entire BLS spectrum simultaneously. A VIPA is a type of Fabry-Perot etalon in which the upstream partial reflector is replaced by a high reflector, except for a small window through which the light is coupled. The dispersion of a VIPA is the same as that of a standard angularly-dispersed etalon. While the latter has a throughput on the order of inverse finesse, the transmission of a VIPA approaches unity for a near diffraction-limited input along the dispersion axis (e.g. confocal collection or line scan geometry)[101]. Consequently, depending on the excitation conditions and sample, VIPA spectrometers can acquire a Brillouin spectrum in a few tens of milliseconds. A FWHM resolution of a few 100 MHz and a sub-10 MHz precision are typical. The VIPA thickness can be determined within 0.1%, facilitating accurate absolute calibration. The simplest VIPA spectrometer setup consists of a collimated beam focused with a cylindrical lens into the entrance window of a VIPA, followed by an imaging lens and a camera to capture the spectrum. The contrast of a single VIPA being inherently limited to ~30 dB, several cross-dispersion configurations and elastic peak suppression strategies have been developed as described below.

Crossed VIPAs: To increase the contrast of spectrally-dispersive optical elements, cross-axis cascading of multiple elements[102] offers an efficient option that has been used in many VIPA-based spectrometers. The principle of cross-axis cascading with VIPA etalons is straightforward: if a first stage of optical dispersion is aligned along a vertical direction, then the spectral pattern is also dispersed vertically. In this situation, any elastic scattering component beyond the contrast of the first spectral stage appears as a crosstalk signal along the vertical spectral axis. If a second stage of spectral dispersion is now aligned orthogonally to the first stage, the spectral pattern exiting the first stage enters the second etalon through the input window. Since both etalons disperse light in orthogonal directions, the overall spectral axis of the double-spectrometer lies along a diagonal direction, which is where the

Brillouin spectrum appears. Instead, the crosstalk elastic signal, which was spatially overlapped after the first stage, after the second stage, is mostly confined to the horizontal and vertical axis. This procedure has been shown to be repeatable for multiple stages[103], but the double-stage configuration has proven to provide the best tradeoff between throughput and contrast. In most realizations, the elastic scattered light is physically blocked with an optical mask in an intermediate image plane or planes to avoid detector saturation. The crossed-VIPA architecture is compatible and often used with other improvements that have been demonstrated over the years such as apodization[104], coronography (Lyott stop)[105] or diffraction masks[106], leading to high-throughput contrast of ~80 dB. These combinations usually suffice for measuring e.g. isolated biological cells, but not for more opaque samples.

Crossed VIPA-echelle grating: Alternatively to crossed VIPAs, contrast enhancement through cross-dispersion can also be achieved with an echelle grating. The latter displaces the Brillouin signals away from the VIPA Lorentzian tails of the typically much stronger elastic signal. This results in a contrast on the order of 50 dB at the water BLS peak position and up to 60 dB beyond. The grating also separates otherwise overlapping VIPA orders, producing an unambiguous simultaneous range of several THz with minimal spectral artifacts. This enables the BLS and ultra-low frequency Raman measurement of a large range of samples in a snapshot. Since this configuration produces a 2D pattern on the camera, calibration involves mapping which pixels can be illuminated and determining to what wavelength they correspond based on the VIPA and grating dispersion equations. Commercial solutions are available employing such a crossed VIPA-echelle grating scheme, which when combined with an elastic suppression filter can achieve an effective contrast of 120 dB[107]. Alignment of the crossed VIPA-echelle grating scheme is similar to a single VIPA, since the grating is essentially alignment-free.

When publishing measurements on VIPA-based spectrometers additional parameters that should be reported are the VIPA FSR, the spectral interval measured on the detector array, the number of orders used to analyze the BLS peaks, and if relevant the model number in the case of a commercial instrument.

Elastic suppression filters: An alternative solution to cross-dispersion that has been used to gain visibility of the BLS spectral peaks, in primarily VIPA-based spectrometers, is through the employment of special high-rejection narrow-band optical filters to attenuate the elastic scattering light. In this case, the elastic background light is suppressed by a notch filter, relaxing the need for multiple optical passes and/or cascaded dispersive elements to achieve high spectral contrast at detection. Employing a sufficiently effective filter can allow one to employ only a single VIPA spectrometer to perform measurements on turbid media.

Several methods have been demonstrated to efficiently suppress the elastic background light with extinction ratio ranging from 30 to 50 dB. Commonly employed methods include the use of etalons[108,109], absorption cells[110] or interferometric schemes[111,112]. Two popular filter types currently being employed are:

Gas absorption cell filters: Atomic and molecular line absorption filters can provide an efficient approach to suppress the elastic scattering in BLS microscopy. Since some of the most commonly used laser wavelengths for BLS microscopy (532 nm and 780 nm) are close to molecular absorption lines of molecular Iodine (532 nm) and atomic Rubidium (780 nm), the use of absorption lines from these to suppress elastic scattering can be used. Here the incident laser light can be tuned match a selected absorption line and be actively stabilized and frequency locked to this line[113]. The advantages are the relatively low cost and easy maintenance of such a filter, which are independent of environmental conditions and beam divergence. The Doppler and collisional linewidth are insignificant for most practical conditions, and a high degree of attenuation can be achieved using multipass geometry or longer cell pathlengths[35,114].

Potential drawbacks of gas absorption cells include the presence of a number of absorption lines that may modify the transmission spectrum and thus final measured spectra (particularly the case in Iodine cells), residual emission from the cell, and a potentially large variation of transmission for a cell whose temperature is not actively stabilized. Some of these issues can be addressed through BLS measurements taken at multiple excitation wavelengths, at the expense of making the overall setup more complicated and less suitable for rapid acquisition[115]. Gas absorption cells also ideally require the use of laser lock-in scheme, which typically comes with an increased optical system complexity. When using gas absorption cells, it is recommended to report the chemical composition of the cell and its dimensions, the number of passes through the cell, the manufacturer and model of the cell, and the temperature of the cell when in use.

Etalon-based filters: A low-insertion loss, ultra-narrow notch filter can be achieved with a Fabry-Perot etalon tuned (angle, temperature, pressure, or piezo) to transmit the excitation wavelength while reflecting the Brillouin signals. The transmitted excitation wavelength light is then discarded, while the light covering the BLS peaks is coupled into the BLS spectrometer, typically via a fibre or small pinhole for spatial filtering. The alignment consists of optimally centering the filter transmission at the laser wavelength, and subsequently optimising the coupling into the output fibre or pinhole. Commercial solutions, by the company Light Machinery, based on a 4-pass pressure-tuned etalon which produces up to 70 dB suppression are available. Other types of interferometers can be used to filter out the elastically scattered light, such as modified Michelson interferometers[112] or prism-based interferometric filters[116].

While the development of integrated filters is ongoing[117], a common-path birefringence-induced phase delay (BIPD) filter providing up to 60 dB extinction ratio in a single pass has been recently proposed[65], and made commercially available by the company *Specto Srl*. This BIPD filter can be optimized for all visible wavelengths without the need of sophisticated laser lock-in systems.

In general, when using such elastic filter(s) one should report their FSR, the type and arrangement of the filter(s), their experimentally obtained extinction, and the manufacturer(s) and model(s) of any elements employed in their realization.

Radial VIPA: Recently the concept of the VIPA has been extended by replacing the entrance *slit* of the VIPA with a *hole*, effectively changing the Cartersian degree of freedom the conventional VIPA offers (which is exploited in e.g. Line-scanning BLS[82,83]) to a radial one[15]. With a suitable excitation scheme this allows one to instantaneously measure (image) the frequency shift, and thereby acoustic speed and longitudinal modulus, in different (azimuthal) directions in a sample and determine the mechanical anisotropy. Being by construction limited to a single pass/VIPA configuration, it requires good suppression of the elastic scattering (as may be achieved by e.g. absorption cells—see above), and currently still only suited to reasonably transparent samples. For dynamic measurements and measurements with low (physiologically acceptable) laser exposure it is necessary to perform angular binning to obtain statistically useful spectra. In addition to reporting the typical parameters for VIPA spectroscopy such as the FSR, and that of elastic scattering suppression schemes described above, it is also important to report the range of wavevectors/scattering-angles probed in the sample (determined by the optical setup before the spectrometer) and the azimuthal and polar angles that are being averaged over for each spectral projection.

Stimulated BLS

Stimulated Brillouin scattering (SBS) is a nonlinear phenomenon driven by the interaction between optical and acoustic fields through electrostrictive, absorptive, and photoelastic processes under energy and momentum conservation conditions. In SBS, induced density variations (i.e., an acoustic wave) in the medium are typically driven by the interference of two incident optical waves which are detuned in temporal frequency and/or spatial frequency around the BLS resonances of the medium. The stimulated acoustic wave can then be observed in the backward direction as an increase (or a decrease) in the intensity of a weak optical wave or can be interrogated in the forward direction by a third readout wave[118]. There are currently two types of BLS microscopy that are referred to as SBS microscopy (*Frequency domain* SBS and *Impulsive* SBS), although Time-Resolved Brillouin Scattering (TRBS) also technically falls into this category (and is thus included here).

SBS microscopy's major limitation is the complexity of the experimental arrangement and the difficulty of the alignment of counterpropagating beams and scanning one of the frequencies. In its earliest implementation[119], the primary challenge came from the signal strength limited by the allowed pump power; however, through pulsed excitation[120,121] and quantum-enhanced imaging[122] the problem of high average power lasers can be largely mitigated. Alternatively the laser exposure can be reduced significantly by only measuring at discrete frequencies[84].

Frequency domain SBS

In frequency domain SBS, two counter-propagating, frequency-detuned optical fields interfere in the medium[26,123]. One field is relatively intense (pump) and the second is typically weaker (probe). The wave interference induces travelling density variations (i.e. an acoustic wave) in the medium primarily via electrostriction. This consecutively produces a travelling refractive index variation through photoelasticity. Under proper incidence polarisation circumstances and frequency detuning that closely matches Brillouin lines of the medium (on the order of 5 GHz at near infrared wavelengths), the pump intensity is reflected from the moving grating with a Doppler shift of the probe light. As a result, the intensity of the probe increases (decreases) with a fractional gain (or loss). The corresponding lifetime of the acoustic wave is ~1 ns at near infrared wavelengths, resulting in BLS linewidths of several hundreds of MHz. When publishing SBS results it is additionally important to report the frequency range over which the probe laser is scanned and the corresponding frequency steps.

Impulsive SBS

In Impulsive Stimulated Brillouin Scattering (I-SBS), two pulses interfere to stimulate acoustic phonons within the sample. The readout process involves Bragg diffraction of a probe beam, commonly a CW laser at a different wavelength, which results in an intensity-modulated time-signal[85,124]. Unlike above mentioned techniques, I-SBS measures in the time domain, allowing for potentially higher time resolution, albeit at reduced spatial resolution. The excited acoustic frequency (f) in I-SBS can be calculated via $f = 2V/d$ f = 2*v / d (for electrostriction), where V is the sound speed, and d is the interference fringe spacing. The frequency is tunable by adjusting the intersection angle θ of the pulse beams. The frequency resolution of I-SBS, or the ability to distinguish between two frequencies in the spectrum, is inversely proportional to the signal length.

The signal length depends predominantly on the size of the excitation volume. A high pulse energy is required to generate high SNR, but requires a good balancing with pulse length and repetition rate. When publishing SBS results it is thus additionally important to report laser pulse characteristics. The interference fringe spacing of the two excitation beams acts as a lower limit for the spatial resolution, because the probe beam has to diffract at least one acoustic period. One possible application of I-SBS can be *Brillouin cell cytometry*[125] where a typical spot size of 10 µm is employed.

Time resolved BLS

Time-domain Brillouin Light Scattering (TRBS), also referred to as *Phonon Microscopy* in the literature, is a collection of techniques that are generally characterised by the following two conditions: (a) there is a photoacoustic generation of coherent acoustic phonons using a pulsed pump laser and a transducer (typically a thin metallic film). (b) One measures the

temporal modulation of the intensity of a second laser beam probing the propagation of the generated phonon, that is representative of the phonon time-of-flight[64,126].

Compared with other BLS imaging techniques, time-domain is capable of spatial resolutions down to the acoustic wavelength, and also provides access to the instantaneous phase of the acoustic wavefront. To construct a time-domain BLS optical system, it is important to consider the 3D spatial overlap of the pump and probe beams. For the initial stages of alignment, it is most convenient to use the top surface of the opto-acoustic transducer as the reference plane. The pump laser should be collimated entering the back aperture of the objective lens. Due to the incompatibility of shearing interferometers with ultrafast pulsed lasers, collimation is verified by visually inspecting the beam size at infinity while adjusting the length of the beam expanding lens pair. The position of the brightfield camera can then be optimised such that the sample and pump beam are both in focus. Next the process is repeated for the probe beam to match the focal plane of the pump and brightfield optics.

The transient reflectivity or transmissivity of the probe beam is typically detected by a ~5.5 MHz bandwidth amplified (10dB) photodiode, further amplified (24dB), and low-pass filtered (11 MHz) before digitisation by an oscilloscope. The time signal on the oscilloscope should be maximised in amplitude by adjusting the x, y, and z overlap of the pump and probe beams. Once maximised the signal can be processed according to the protocols set out in[92] to extract the phonon time-of-flight, which should be normalised by the DC light level (equivalent to the amplitude of the Rayleigh peak) to achieve units of modulation depth. This processed modulation depth signal can be analysed in the frequency domain through Fast Fourier Transform to measure the BLS frequency shift ν_B. The phonon attenuation rate is related to the longitudinal viscosity and can be measured directly in the time-domain by fitting a decaying exponential to the TRBS signal. The decay constant (α) is typically interpreted in units of μm^{-1} by converting the time-base of the signal into the axial spatial domain through the inferred relationship between the BLS frequency shift and sound velocity. However, in order to provide an equivalent BLS linewidth measurement in the frequency domain (after taking an FFT of the time-signal), the finite nature of the time window must be considered as this introduces a spectral artifact in the form of the Fourier transform of a Heaviside function (truncation of the time window). This can be calculated analytically and results in the following correction formula: $\Gamma_B \approx \Gamma_{TRBS} - 3.78/(2\pi \mathrm{T})$ where T is the length of the time window and Γ_{TRBS} is the full width half-maximum of the Brillouin spectral peak.

It is recommended to report the modulation depth and SNR of a given time-domain BLS setup, along with experimental parameters such as the average optical power, NA of the illuminating objective lens, laser repetition rate, and pulse width. The modulation depth scales with the acoustic amplitude (proportional to pump intensity) and probe intensity: $I_{pump}I_{probe}$. Consequently, the specimen type will dictate the sustainable pump and probe intensities (and resulting transducer heat rise). Utilising sapphire substrates and pump and probe powers of 1 and 3 mW respectively, a modulation depth of ~10^{-4} and SNR of ~70 can be obtained[64] using a transducer with 20:160:20 nm Au:ITO:Au layers. For living cells less power should be used (0.5mW pump and 1mW probe) and typically modulation depths on the order of 10^{-6}

and SNR of~40 are obtained with transducer heating on the order of ~1°C[64,127]. To estimate the SNR of a time-domain signal, the amplitude of the BLS peak is divided by the standard deviation of amplitudes in the band of interest from an additional measurement with the pump beam blocked. The SNR will scale proportionally to the pump and probe intensities, SNR $\propto I_{pump}\sqrt{I_{probe}}$ with typical values in the range of 40-70.

Accurate measurement of the BLS frequency shift from time-resolved Brillouin scattering (TRBS) signals can be affected by artefacts generated from several signal components. When both pump and probe pulses overlap in time, optical absorption induces a strong electronic excitation within the metallic transducer and results in a sharp signal response known as the coincidence peak. This manifests itself as a rapid change in the optical properties, temperature, and ultimately the propagation of coherent phonons. The response typically relaxes according to the thermal diffusivity of the system before the arrival of the next coincidence peak. Once the sample volume has been excited mechanically there are additional frequency components in the signal which should be digitally filtered out. The mechanical resonance of the transducer (~10GHz) in sapphire decays after only one or two cycles but it can be more prominent on softer substrates; and the BLS frequency shift of the substrate material which for stiff materials exceeds the detection bandwidth of the system can be digitally low-pass filtered.

To isolate the TRBS signal from these components, the following digital operations may be performed in post-processing[128]: cropping the signal after the coincidence peak, low-pass filtering, and subtraction of the thermal background via low-order polynomial fitting. Cropping too close to the coincidence peak can cause reduced SNR and BLS frequency shift measurement precision due to the presence of broadband signal components, and cropping too far artificially attenuates the desired signal. Similarly, a polynomial fit order that is too low can cause errors in the determination of the BLS frequency shift, whereas an aggressive polynomial can act as a high pass filter.

The frequency resolution of the system is given by the repetition rate of the lasers (80-100MHz) which is typically insufficient to detect small variations in the BLS frequency shift in biological tissue. However, since the bandwidth of the signal is much greater than the frequency bin spacing, the spectrum can be interpolated by zero padding before calculating a Fourier transform (2^{14}-2^{16}), allowing one to determine the centre frequency of the spectrum with ~10 MHz precision[92]. In the case of estimating the longitudinal sound attenuation coefficient, it is important to note that a short optical depth of focus at high NA can lead to measurement errors. This is due to the signal decaying by loss of optical intensity rather than acoustic intensity. The acoustic attenuation in water at 5.1 GHz and at room temperature is ~$2.5\times10^5 m^{-1}$, and comparing deviations to this value can provide a means for correction[29].

In time-domain BLS the photo acoustically-stimulated coherent phonon field occupies a well-defined region in space, defined laterally by the PSF of the pump laser at the transducer interface, and axially by an exponentially decaying envelope set by the phonon path length,

and with a well-defined wavevector (approximately a plane wave). It follows that the fundamental limit for lateral resolution is defined by the convolution of the spatially overlapped pump and probe PSF. Since the measured time of flight of the scattered probe beam is proportional to the axial distance, its period is that of the acoustic wavelength (which is shorter than the optical wavelength by a factor $\lambda_{optical}$/2n), and time-frequency analysis allows for measurements of ν_B with an axial resolution down to that of the acoustic wavelength[91,127]. Conceivably the lateral spatial resolution can also be reduced to that of the phonon wavelength if one were to employ opto-acoustic lenses and time of flight analyses.

Some parameters important to additionally report in TRBS are the modulation depth and SNR of the TBBS setup, as well as the repetition rate and pulse width of all employed lasers.

Heterodyne BLS

Heterodyne detection is a potent technique utilised in various optical measurements, including BLS. In standard BLS detection, the measured signal is a measure of the number of photons collected within a given bandwidth and integration time, that is proportional to the square of the scattered electric field. Heterodyne detection entails mixing the scattered light with a reference beam, termed the local oscillator (LO), at a slightly different frequency. The interference between the two beams generates a beat frequency in the radiofrequency (RF) range, which is linear with the weak BLS electric field and the strong LO field. This setup often operates with LO power around 1 mW and scattered light power in the femto-Watt range, shifting the challenge from low light optics to low electric signals in the sub-GHz region.

An analog heterodyne detection setup[129] typically includes several key components. A monochromatic light source passes through the sample, where it undergoes BLS. The scattered light is then mixed on a square law photo-detector with the LO beam. A microwave driven electro-optic amplitude modulator generates the LO by shifting the source at specific frequencies close to the Brillouin peak. This brings the beat frequency into the sub GHz range, where the two electric signals are amplified, filtered using a bandpass filter, and further cleaned up by a lock-in based detection. The width of the bandpass filter determines the spectral resolution of the BLS measurement.

A digital heterodyne detection setup[130], on the other hand, incorporates high-speed digitizers and Field-Programmable Gate Arrays (FPGA) for data processing. Similar to the analog setup, the scattered light and EOM generated LO beam are mixed, producing the beat frequency. The digitizer samples the beat signal at high rates, such as 3.2 Gs/s, and the FPGA processes the data in real-time. This digital approach offers higher processing speed and flexibility, enabling real-time data analysis.

Heterodyne detection is an intrinsically single mode measurement, thus the usual single mode fibre collection of Brillouin microscopy confocal layout is best suited for this approach.

The strength of this approach is that, thanks to the down conversion of the optical heterodyne mixing, contrast and resolution are no longer a major issue, as they are not limited by the transfer function of optical devices, but benefit from the tools and precision offered by electronics in the sub GHz region. As a consequence the approach promises a calibration free, extremely stable and compact layout.

When using a heterodyne detection setup it is additionally important to report the manufacturer(s) and model(s) of instruments used, the achieved spectral resolution , as well as characteristics of the bandpass filter and when relevant those of the Analog to Digital Converter (ADC).

Fibre probes

BLS fibre probes are devices made of or compatible with optical fibre components that can replace the confocal microscope ("front-end") of a BLS imaging system. Different designs have been proposed and demonstrated to date based on whether signal acquisition occurs in the frequency or time domains.

For the frequency domain, BLS fibre probes can be constructed using a single or dual optical fibre configuration and then supplemented by a standard VIPA or a scanning TFP spectrometer to produce a fibre-integrated BLS measurement setup. The main drawback in using optical fibres for delivery of the laser light and for the collection of the backscattered light, is the spontaneous BLS that occurs inside the fibre core. Since light is tightly focused inside the core of the optical fibre, and a typical length of an optical fibre is a few metres, the effective interaction volume between light and fibre core material is significantly greater than the volume of the optical voxel formed by the focussed beam in the sample. Thus, despite the fibre BLS frequency shift usually being significantly larger than that of the probed sample (>20 GHz), this parasitic signal might present a challenge in detecting the BLS signal originating from the sample[35]. Different solutions to this problem exist, including: 1) hollow-core optical fibres for delivery of the laser illumination to the sample without inducing any significant Brillouin scattering in the fibre itself[88]; 2) the use of dual-core or dual-fibre geometries, as is done in Raman endoscopy, where delivery and collection channels are separated thus avoiding mixing of the fibre backscattering signal with that from the sample[87]. Dual fibre approach requires careful design of the focusing optics in order to ensure collection of the inelastically scattered light back into the core of the second fibre. This can be done by creating on demand 3 dimensional microoptics and/or compound lenses fabricated via two-photon-polymerisation laser writing technique. Overall, the imaging resolution in lateral and axial directions depend on the focusing micro-optics design and is typically slightly worse when compared to conventional microscopy objective lenses, owing to low numerical aperture of the single-mode optical fibre and fibre-compatible lenses[88].

Brillouin fibre probes can also be constructed to operate on the principles of TRBS; however this requires fabrication of an optoacoustic transducer onto the fibre tip for the generation of

coherent phonons. These stimulated phonons provide an enhanced BLS cross-section, however currently at the cost of slower detection electronics. The time-domain BLS process is largely insensitive to BLS back scattering along the glass fibre - since the high amplitude stimulated phonons are localised to within 10-20 μm of the transducer as is the coherence length of the interaction. This means that standard silica fibres of arbitrary length can be used.

A time-domain BLS system can be converted for fibre implementation by fibre coupling the free-space pump and probe beams and coupling these into a common channel via a 2:1 fibre coupler. The common channel contains an inline fibre circulator, containing the sample-facing fibre at one port and the detection photodiode at the other, thus avoiding losses incurred by using standard 3dB couplers. If the pump and probe beams contain separate wavelengths, then SNR can be further improved by using a wavelength division multiplexer to attenuate the pump beam at the detector and reduce its contribution to shot noise[92]. SNR and modulation depths will be comparable to free-space TRBS provided similar parameters are used for the optical system and optoacoustic transducer.

Time-domain fibre probes have been shown can achieve 2 μm lateral resolution, realised by point-scanning the sample or fibre, and down to 260 nm depth resolution (equivalent to the phonon wavelength) which is achieved without confocal scanning the optics since the time-domain signal encodes the depth domain[91]. However, high depth resolution comes at the cost of reduced depth measurement range (~10 μm) which can be increased by lengthening the probe wavelength (the attainable depth scales as the square of the probe wavelength).

The exact details of the fibre inelastic scattering depend on the type of fibre used, its length, the amount of bending of the fibre, and other properties of the experimental configuration. Even the use of hollow-core optical fibres does not remove the fibre background completely since a small percentage of the fundamental fibre mode and higher order modes interact with the microstructured region of the hollow-core fibre, producing a small amount of backwards-scattering BLS[88]. Such a parasitic signal, however, does not interfere with the desired BLS signal from the biological sample, as it is positioned outside the spectral region of interest, and can be removed during data post-processing. High order fibre modes may also be attenuated by bending the hollow-core fibre slightly, without inducing significant bending losses to the fundamental fibre mode.

As with spontaneous BLS fibre probes, in TRBS fibre probes, the BLS signal from the glass fibre is typically low-pass filtered in post-processing. It is worth noting that the time-domain BLS process is only sensitive to the stimulated coherent phonons within the vicinity of the transducer, and although these are approximately 6 orders of magnitude greater amplitude than the spontaneous thermal phonons along the fibre length, they attenuate very rapidly, travelling away from the transducer within tens of microns. This allows one to use long lengths of glass fibre without adversely affecting the glass signal amplitude. Additionally, the acoustic impedance and photoelastic coefficient mismatches between the glass, metal and water layers result in a ~2 orders of magnitude greater signal amplitude from the water or cell tissue interface compared to that from the glass interface. Sample heating should be considered

when performing fibre-based time-domain BLS both in terms of specimen viability and changing the measured BLS frequency shift. The former can be mitigated through lowering optical fluence and the inclusion of thermally conductive transducer layers. Any thermal gradient produced by the fibre probe can be calibrated by obtaining reference measurements (away from a specimen) in the aqueous couplant medium. These have been found to be stable under normal experimental conditions, such that subtracting these frequency offsets from the specimen BLS shifts can potentially compensate for such thermal contributions[91,92].

When using fibre probes it is additionally important to report the manufacturer and model of the optical fibre, any strategies used to overcome the contribution from BLS backscattered light from the fibre, the coupling efficiency of reflected and/or Brillouin scattered light back into the fibre-core prior to detection, and as relevant any treatment performed on the tip of the fibre. When using transducers at the end of fibre probes, it is additionally desirable to present an assessment of the sample heating induced by the transducer, and any relevant calibrations made for this.

2: Some useful equations for BLS microscopy

The interpretation of Brillouin Light Scattering (BLS) measured parameters is historically divided between formalisms that consider the measured parameters in two different frameworks.

Firstly, there is that of acoustic (e.g. shock) waves moving through a solid and largely elastic material, which is relevant for describing e.g. geological activity, mechanical properties of polycrystalline materials, etc.. Here the material properties are often treated as effective entities, with the interest typically being how the collective, and for the most part elastic, properties change e.g. in different directions. Given the characteristic relaxation times of *hard* solids are very slow, the BLS derived *stiffness coefficients* can be useful for modeling also lower frequency acoustic and mechanical properties.

Secondly, there is the thermodynamic and (molecular) hydrodynamic picture, which considers the molecular interactions on short time scales to predict the supported phonon modes. From this one can gain insight into the electrostatic screening of polymers (such as DNA) and structural states of molecules that affect their interactions with a solvent or molecules in their immediate vicinity. Here the time scales probed using BLS may become more comparable to those of the relaxation processes, and one can gain insight into the nature of e.g. phase and structural transitions by virtue of the changes in the hypersonic speed and attenuation. The interpretation here is less on our everyday understanding understanding of viscoelastic moduli, but rather on elucidating changes in “viscoelastic properties” on time scales that affect the molecular interactions, namely relaxation times, and how these may lead to changes in both molecular processes and collective material properties.

Biological systems, such as a living cell, rarely fit either of these two, but rather often exist in a somewhat undefined dynamic state between an anisotropic solid and a liquid, usually containing elements of each. In regard to building on the established BLS nomenclature this presents a unique challenge. Below we define some parameters, taken from these two established branches, that can prove useful for analyzing BLS measurements in biological systems. These include both basic fitting parameters and biophysically relevant parameters, with a brief justification of their relevance in each case. These are intended to serve as a potentially useful, but in no way complete, reference for people performing BLS on bio-relevant matter, to assure that all parameters are defined consistently.

Fitting equations

Damped Harmonic Oscillator: In the frequency domain, the intensity of each BLS peak is functionally described by a Damped Harmonic Oscillator (DHO) given by:

$$I(\nu) \propto \frac{\Gamma_B \nu_B^2}{\left(\nu^2 - \nu_B^2\right)^2 + (\Gamma_B \nu_B)^2} \qquad \textbf{Eqn. S1}$$

where ν is the frequency shift relative to the probing laser frequency, and ν_B and Γ_B are the BLS frequency shift and BLS linewidth. If ν_B falls in the vicinity of a structural relaxation processes ($\sim 1/\tau$, where τ is the viscous relaxation time) the shape of the peak will generally have a more complex form, and its spectral shape needs to be calculated from first principles[1,22].

Lorentzian Function: To an, often good, approximation the BLS peak can also be described by a Lorentzian function:

$$I(\nu) \propto \frac{\Gamma_B^{(L)}}{\left(\nu - \nu_B^{(L)}\right)^2 + \left(\Gamma_B^{(L)}/2\right)^2} \qquad \textbf{Eqn. S2}$$

where $\nu_B^{(L)}$ and $\Gamma_B^{(L)}$ are the (Lorentzian) BLS frequency shift and BLS linewidth. If the linewidth is not too large this is usually a good approximation.

Correction to Lorentzian fit: Due to the general asymmetry of the DHO, the Lorentzian fitting parameter $\nu_B^{(L)}$ will differ from the true (DHO obtained) ν_B by an amount that becomes more significant with increasing linewidth given by:

$$\nu_B = \nu_B^{(L)} \sqrt{[1 + (1/2)\Gamma_B^{(L)}/\nu_B^{(L)}]} \qquad \textbf{Eqn. S3}$$

Frequency shift

The frequency shift ν_B of a given BLS peak can be expressed in terms of the hypersonic speed V_q of the respective measured phonon modes in the direction of the scattering wavevector q:

$$\nu_B = (2\pi)^{-1}\omega_B = \pm(2\pi)^{-1} q V_q \qquad \textbf{Eqn. S4}$$

The scattering wavevector *q* (which is also the wavevector of the created/anihilated phonons) is given by:

$$q = 4\pi n \sin(\theta/2)\,/\lambda_0 \qquad \textbf{Eqn. S5}$$

Here θ is the scattering angle (=180 degrees in the back-scattering geometry), n is the refractive index, and λ_0 is the free-space wavelength of the probing laser.

In the vast majority of BLS microscopy studies on biological samples (and to date practically all studies on living cells) one measures only the *longitudinal acoustic phonons*. These in almost all cases have larger BLS frequency shifts than the transverse phonons also supported in solids and some liquids, and a much larger scattering cross section. They correspond to travelling density waves where the displacement of molecules is entirely in the direction of the propagating waves. Namely, solutions of the wave equation:

$$\frac{\partial^2 u_i}{\partial t^2} = V^2 \frac{\partial}{\partial x_j}\left(\frac{\partial u_l}{\partial x_k}\right) \qquad \textbf{Eqn. S6}$$

in which u_i is the displacement of molecules in the direction x_i, and t is time, with $i = j = k = l$. The speed V here is related to the mass density (ρ) of the material, as well as its stiffness. The relevant stiffness here depends on the direction of the strains relative to the stresses. These can be expressed in terms of the generalized Hooke's Law:

$$\sigma_{ij} = c_{ijkl}\varepsilon_{kl} \qquad \textbf{Eqn. S7}$$

Where $\sigma_{ij}$and ε_{kl} are the strain and stress tensor (and the subscripts indicate the decomposed orthogonal vector directions, i.e. $i = 1,2,3$). c_{ijkl} is the so-called *stiffness tensor* and describes the complete elastic response of a material in an arbitrary direction subject to a stress in the same or a different arbitrary direction. $c_{ijkl}$is a huge tensor, but owing to several symmetry constraints only has 21 independent components and is usually more compactly expressed as a 6 x 6 tensor c_{ij}. The first three diagonal components (c_{11}, c_{22} and c_{33}) will correspond to longitudinal modes (i.e. where $i = j = k = l$) in the three orthogonal directions.

In general the relation between the hypersonic speed in the direction of the probed scattering vector $\hat{\boldsymbol{q}} = \boldsymbol{q}/|\boldsymbol{q}|$, and the relevant stiffness tensor components can readily be obtained from the Christoffel equation:

$$\left|(\hat{\boldsymbol{x}}_{\boldsymbol{k}} \cdot \hat{\boldsymbol{q}})(\hat{\boldsymbol{x}}_{\boldsymbol{l}} \cdot \hat{\boldsymbol{q}})c_{ijkl} - \rho V_q^2\right| = 0 \qquad \textbf{Eqn. S8}$$

where $\hat{x}_i$ are unit vectors in the direction of the respective subscripts. It follows that the speed of the longitudinal phonons will in the direction $\hat{x}_i$ be given by:

$$V_i = \sqrt{(c_{ii}/\rho)} \qquad \textbf{Eqn. S9}$$

where c_{ii} describes the response to a stress and strain in the same direction (i).

The longitudinal elastic modulus will normally correspond to the stiffness tensor components c_{11}, c_{22} or c_{33} when the material is measured in a symmetry direction such as along a symmetry axis for a crystal, along the fibre axis for a fibre, or in essentially any direction for an isotropic material like an amorphous material, gel, or liquid. Conceptually the longitudinal elastic modulus can be thought of as the relevant modulus when the strain is uniaxial. This means that the material is stretched or compressed only in a single direction and does not change in the perpendicular directions.

If one assumes that the material is mechanically isotropic (has same elastic modulus in all directions) then $c_{11} = c_{22} = c_{33} = M'$ and it follows from Eqn. S4, S5 and S9 that:

$$\nu_B = 2n\,\lambda_0^{-1}\sqrt{\frac{M'}{\rho}}\,\sin\left(\frac{\theta}{2}\right) \qquad \textbf{Eqn. S10}$$

which is the key equation used to calculate the longitudinal elastic modulus from BLS frequency shift measurements. It is important to note that this is for a single scattering angle (θ), whereas one in practice measures over a finite range of scattering angles (on account of the finite NA of the microscope objective), which will result not only in peak broadening but also potential shifts in ν_B. To this end one should ideally integrate Eqn. 1 over the solid probing and collection angles, especially for scattering geometries deviating from back scattering, to obtain accurate measures of M'.

The above assumes that not only the longitudinal elastic modulus but also that the refractive index (n) is isotropic. The latter may not always be the case, e.g. in fibrillar structures such as muscles or cellulose. In such cases its directional dependence needs to explicitly be accounted for, which may be done by writing it in the form of a tensor ($n \rightarrow \boldsymbol{n}$) and considering its projection in the direction of the scattering wavevector. The BLS frequency shift can then be compactly expressed as:

$$\nu_B = \pm 2\,\boldsymbol{n}\cdot\hat{\boldsymbol{q}}\,\lambda_0^{-1}\,V_q \qquad \textbf{Eqn. S11}$$

Where V_q in a mechanically anisotropic sample is in general a combination of stiffness tensor components defined by the projection of the scattering wavevector that can be calculated from Eqn. S8.

It is also possible to measure BLS scattering from transverse acoustic phonons. These show up as peaks at (usually) lower frequency shifts than the longitudinal phonon peaks and typically require the implementation of distinct (non-backscattering) measurement geometries. From these it is possible to calculate the *shear modulus* (G) (also called the *transverse modulu*s), with the real part (G') obtainable from the peak frequency shift in an

analogous manner to the longitudinal elastic modulus using Eqn. S4, S5, S9 and S10. The shear modulus is a stiffness coefficient (c_{ij}) that describes cases where the strain is pure shear. This means that the material's shape is changed but its density is not. It is the appropriate modulus for transverse sound waves. As with the longitudinal modulus, it can be different in different directions unless the material is isotropic. It will normally correspond to c_{44}, c_{55}, or c_{66} of the stiffness tensor when measuring in a symmetry direction in a crystalline material. Typically two of these three coefficients show up as two distinct BLS peaks in a single measurement. These two peaks correspond to the two principal polarizations of the transverse phonon mode. The transverse modes are usually measured in distinct (90 degree) scattering geometries, although in anisotropic systems they can also be revealed in back-scattering measurements, provided that the wavevector is not along a direction of high symmetry. Because they are generally isotropic, amorphous materials will only have a single shear modulus.

Linewidth

The BLS linewidth (Γ_B), i.e. the Full Width at Half Maximum (FWHM) of the BLS peak, obtained from fitting a DHO or Lorentzian function (Eqns. S1 & S2), is fundamentally determined by the lifetime of the phonon--in an analogous manner to the peak width in a high energy physics experiment, with the (quasi-)particles in this case being the acoustic phonons. Given the phonons propagate at a presumed speed V_q , this lifetime is directly related to their spatial attenuation length. It is possible to also calculate a corresponding *loss modulus* that describes the dissipative properties of the sample (i.e. the corresponding imaginary part of the stiffness tensor components). In the case of longitudinal phonon modes this would be:

$$M'' = 2\pi\rho q^{-2}\nu_B\Gamma_B \qquad \textbf{Eqn. S12}$$

For a mechanically isotropic sample, M'' is the imaginary part of the *complex longitudinal modulus*: $M = M' + iM''$, and $i = \sqrt{-1}$. But as with the real part, M'' can in general take on different values in different directions (i.e. M''_{ij}). An analogous relation may be obtained from the BLS frequency shift and linewidth of the transverse acoustic modes, yielding the imaginary part of the shear modulus (G'').

For fluids it may sometimes also be desirable to express the dissipative properties in terms of a viscosity. For this the following relation may be used:

$$\Gamma_B = \frac{q^2}{2\pi\rho}\left[\frac{4}{3}\eta_S + \eta_B + \frac{\kappa}{C_P}(\gamma - 1)\right] \qquad \textbf{Eqn. S13}$$

Here η_S is the *dynamic shear viscosity* common to rheology (but in this case that the probed MHz-GHz frequencies). $\eta_B$is the *dynamic bulk viscosity* (also referred to as the second viscosity by e.g. Landau and Lifschitz), which is a distinct material property to the shear viscosity, that in practice can usually be neglected from fluid dynamics calculations, i.e.

Navier Stokes equation, on presumption of the incompressibility of flow. It however becomes relevant when there are e.g. bubbles, one considers microscopic instabilities, or of course for the propagation of longitudinal sound waves. κ is the thermal conductivity of the sample, and $\gamma = C_P/C_V$ where C_P (C_V) are its specific heat under constant pressure (volume). In water based matter one often can assume that $C_P \approx C_V$ such that $\gamma \approx 1$ and that the linewidth is directly proportional to the combination of the dynamic shear and bulk viscosity with no offset:

$$\Gamma_B \approx \frac{q^2 \eta_L}{2\pi\rho} \qquad \textbf{Eqn. S14}$$

Where $\eta_L = (4/3)\eta_S + \eta_B$ is the *dynamic longitudinal viscosity* (sometimes also called the *effective* or *dilational* viscosity in the literature) that is relevant for describing the attenuation of longitudinal acoustic modes. η_L can serve as a good reporting parameter insofar that in a Newtonian fluid under hydrodynamic conditions it will be independent of frequency and wavevector (compared to the loss modulus and acoustic attenuation, which would scale linearly and quadratically respectively with the probing wavevector--and thus also the refractive index). For some applications it may also be relevant to calculate the *kinematic longitudinal viscosity* μ_L, which is defined as $\mu_L = \eta_L/\rho$.

For measurements of transverse phonons, the linewidth can analogously yield information on the imaginary part of the shear modulus (G'') and shear viscosity (Eqn. S12 and Eqn. S14 with the replacement $\eta_L \to \eta_S$ in the latter). The shear viscosity and moduli measured using BLS will be distinct from that measured using classical rheological techniques (shear rheology, etc. which measure at low shear rates) as it is typically at many orders of magnitude higher frequencies.

The above assumes that there are no extrinsic factors that cause broadening of the BLS peak (i.e. increases in the linewidth), and are defined for measurements at a single scattering wavevector. In practice numerous factors will also cause broadening of the BLS scattering peak and over estimations of the linewidth. These include measurements from a distribution of scattering angles (finite NA), material heterogeneities in the probing volume (that are larger than the characteristic length scales of the probed phonons), multiple scattering (most relevant for non-transparent samples or deep tissue imaging), and ultimately the usually non-negligible spectral Instrument Response Function (IRF) of the spectrometer. In addition when the probed phonon frequency is in the vicinity of a structural relaxation process with characteristic time(s) τ, namely $\nu_B \sim \tau^{-1}$, as may happen to be the case in soft-matter/gels, the functional dependence of the BLS scattering peak will undergo significant broadening and deviate from a simple DHO.

While a spectral deconvolution may suffice in correcting for the IRF and finite-NA broadening, accounting for the other factors analytically or even numerically is less trivial, requiring a priori information on the sample that is usually not available. To this end a experiment/sample-specific semi-quantitative (or atleast qualitative) assessment of their expected relative contributions should be made, and caution should be exercised when presenting absolute values of linewidth-derived parameters.

Loss tangent

This can serve as a useful metric of changes in the viscoelastic properties owing to it being independent of changes in the mass density (which are challenging to measure on the microscopic scale in complex biological samples). It is defined, for the longitudinal modes, as:

$$\tan\delta = \Gamma_B/\nu_B = M''/M' \qquad \textbf{Eqn. S15}$$

An analogous quantity may also be defined for the transverse (shear) modes. It is sometimes stated that $\tan\delta$ is independent of the refractive index n. This is only true if both M'' and M' are assumed to be independent of the phonon wavevector probed q (Eqn. S5) (or have the exact same functional dependence thereon). While potentially valid in hard dehydrated solids, this is not the case in the hydrodynamic picture, where $\Gamma_B \propto q^2$ (Eqn. S13) and $\nu_B \propto q$ (Eqn. S4), such that $\tan\delta \propto q \propto n$. In the vicinity of structural and phase transitions this assumption can be assumed to break down even more dramatically.

Relation to other moduli

In an isotropic material, the longitudinal elastic modulus is related to the shear and bulk elastic modulus via:

$$M' = (4/3)G' + K' \qquad \textbf{Eqn. S16}$$

This is true also for the imaginary component (loss moduli), with the longitudinal viscosity being defined analogously: $\eta_L = (4/3)\eta_S + \eta_B$ (see Eqn. S13 and S14).

In a linear, isotropic material the longitudinal modulus (M), Young's modulus (E), Shear Modulus (G) and bulk modulus (K) are related via the Poisson ratio (σ):

$$M = \frac{E(1-\sigma)}{(1+\sigma)(1-2\sigma)}; \; E = \frac{9KG}{(3K+G)}; \; G = \frac{E}{2(1+\sigma)}; \; K = \frac{E}{3(1-2\sigma)}$$

$$M = \frac{1-\sigma}{(1+\sigma)(1-2\sigma)}E = \frac{2(1-\sigma)}{(1-2\sigma)}G = \frac{3(1-\sigma)}{1+\sigma}K \qquad \textbf{Eqn. S16}$$

These equations are only valid $-1 < \sigma < 0.5$, with the material becoming inherently unstable outside of this range. In practice, their relevance will also already break down when E/G and M/K become significantly different as will be the case when σ approaches 0.5. Here one would need to know σ to an increasingly higher accuracy (to an infinite accuracy in the limiting case of $\sigma = 0.5$). It should be kept in mind that the Poisson ratio is also a frequency dependent quantity, and can not generally be assumed to have the same values at MHz-GHz as it does at low frequencies.

While Eqn.S15 and S16 may be used to convert between moduli (assuming one has knowledge of the Poisson's ratio at the relevant MHz-GHz frequencies), the obtained e.g. *E* or G can in the general case not simply be compared to measurements obtained from techniques probing at quasi-static frequencies (AFM, shear rheology, etc.) due to the many orders of magnitude differences in frequency, and often unknown mechanical relaxation spectrum between these frequencies. For specific sample types it may however be possible to realize significant and useful empirical correlations between the different moduli, and even the same or different moduli at different frequencies.

An additional caveat for calculating e.g. *E* from the BLS measured *M* and *G* that may become relevant, is that the BLS measured *M* and *G* may in themselves be at quite different frequencies--namely the BLS frequency shifts measured for the longitudinal and transverse phonons may be a couple of orders of magnitude apart. Eqn.s S15 and S16 on the other hand assume that all quantities are at the same frequency.

Much of the above is based upon the assumption of hydrodynamic behaviour and that there are no significant phase or structural transitions at or near the probed BLS frequencies. This may or may not be the case in biorelevant matter, and this should always be kept in mind.

Finally, a difference in the BLS measurements and perturbation-based measurements of these these moduli may also be due to the latter usually measuring elastic moduli by applying a finite-strain (obtaining the so-called *engineering* stress-strain), whereas BLS probing the isentropic *true* stress-strain.

Calculation of stiffness tensor

It is in principle possible to determine the complete stiffness tensor by performing BLS measurements of the sample with different scattering wavevectors. Measurements of an anisotropic sample in an arbitrary direction that does not correspond to a symmetry axis, will yield a possibly complex algebraic combination of stiffness tensor coefficients (which can be determined by Eqn. S8). Such measurements are relatively uncommon except in cases where the experimenter is explicitly trying to extract the values of all stiffness tensor elements including off-diagonal elements such as c_{12}. Most measurements are usually performed in high symmetry directions, where the effective stiffness corresponds to just a single coefficient from the stiffness tensor, such as c_{11}, c_{22}, or c_{33} for a longitudinal mode or c_{44}, c_{55}, or c_{66} for a transverse mode (see Figure S1).

$$\underset{\text{stress}}{\begin{pmatrix}\sigma_{xx}\\ \sigma_{yy}\\ \sigma_{zz}\\ \hdashline \sigma_{yz}\\ \sigma_{zx}\\ \sigma_{xy}\end{pmatrix}} = \overset{\text{longitudinal}}{\underset{\text{shear}}{\left(\begin{array}{ccc:ccc} c_{11} & c_{12} & c_{13} & c_{14} & c_{15} & c_{16}\\ c_{21} & c_{22} & c_{23} & c_{24} & c_{25} & c_{26}\\ c_{31} & c_{32} & c_{33} & c_{34} & c_{35} & c_{36}\\ \hdashline c_{41} & c_{42} & c_{43} & c_{44} & c_{45} & c_{46}\\ c_{51} & c_{52} & c_{53} & c_{54} & c_{55} & c_{56}\\ c_{61} & c_{62} & c_{63} & c_{64} & c_{65} & c_{66}\end{array}\right)}} \underset{\text{strain}}{\begin{pmatrix}\varepsilon_{xx}\\ \varepsilon_{yy}\\ \varepsilon_{zz}\\ \hdashline \varepsilon_{yz}\\ \varepsilon_{zx}\\ \varepsilon_{xy}\end{pmatrix}} \begin{matrix}\text{longitudinal}\\ \\ \text{shear}\end{matrix}$$

Figure S1. BLS in a symmetric direction measures the blue (longitudinal modes) or red (transverse modes). All other stiffnesses are of mixed quasi-transverse / quasi-longitudinal nature and are found by fitting the BLS signal to the Christoffel Equations.

To measure all the stiffness tensor elements requires measuring the sound speeds along different directions which will correspond to the different stiffnesses (c_{11}, c_{22}, etc.) using a combination of different scattering geometries and rotations of the sample. Some common scattering geometries are shown in Supplementary Table 1. The BLS experiment should be altered depending on the symmetry of the sample. A truly isotropic material only has two independent stiffness coefficients (c_{11} the longitudinal component, and c_{44} the transverse component) and these are easily measured, without needing to know the refractive index, by using an equal angle scattering geometry (90a, see Supplementary Table 1). This can however be complicated in cells and other small materials. A single spectrum in an equal angle geometry will give a longitudinal mode, which gives c_{11}, and a transverse mode, which gives c_{44}. This gives all the stiffness tensor elements for an isotropic material.

Geometry	Schematic	Equation
**180**	180a 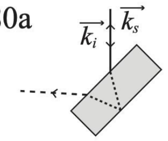	$V_{180} = \dfrac{\Delta\nu_{180}\,\lambda_o}{2n}$
**90n**	90n 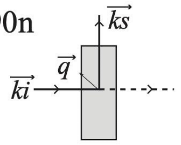	$V_{90n} = \dfrac{\Delta\nu_{90n}\,\lambda_o}{n\sqrt{2}}$
**90a Equal-Angle**	90a 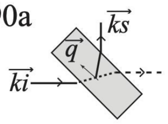	$V_{90a} = \dfrac{\Delta\nu_{90a}\,\lambda_o}{\sqrt{2}}$

**90r**	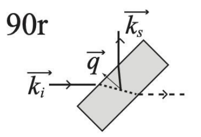	$V_{90r} = \frac{\Delta\nu_{90r}\,\lambda_o}{\sqrt{4n^2-2}}$
**Platelet** (60°)	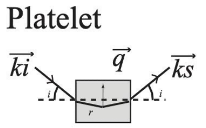	$V_{60} = \Delta\nu_{60}\,\lambda_o$

Supplementary Table 1: Common BLS scattering geometries used for determining the diagonal stiffness tensor coefficients in materials of known symmetries. $k_i(k_s)$ are incident and scattering wavevector, q is the scattering wavevector, and $\Delta\nu$ is the measured frequency shift.

For some applications, one wants to know other elastic properties such as the Young's modulus (E), the bulk modulus (K), the Poisson's ratio (σ), or the shear modulus (G), averaged over crystal orientations. These can all be derived from the stiffness coefficients c_{ij}. For an isotropic material we have three distinct stiffness coefficients c_{11}, c_{12}, and c_{44}, but only two are independent because of the constraint $c_{44} = (c_{11} - c_{12})/2$. The different moduli can then be found from:

$$E = \frac{3c_{12}c_{44}+2c_{44}{}^2}{c_{12}+c_{44}} = \frac{(c_{11}-c_{12})(c_{11}+2c_{12})}{c_{11}+c_{12}}$$

$$G = c_{44}$$

$$K = \frac{1}{3}(c_{11} + 2c_{12})$$

$$\sigma = \frac{c_{12}}{c_{11}+c_{12}} \qquad \textbf{Eqn. S17}$$

As also apparent from Eqn. S17, these equations show that the longitudinal modulus c_{11} is not the same as the Young's modulus but is some combination of the Young's modulus and the Poisson's ratio – expressed in this case as combinations of stiffness tensor elements.

Determining the entire stiffness tensor in a more complex anisotropic sample requires measurements in several directions of the material including off-axis or off-symmetry directions. The experimental design must ensure that the measurements are sensitive to all the stiffness tensor elements and not just the ones along the main diagonal. This means measuring not just pure longitudinal and transverse modes but also mixed modes, so-called *quasi-longitudinal* and *quasi-transverse* mode. BLS frequency shifts are measured as the material is rotated from one on-axis direction to the next, thus rotating the direction of the phonon wave-vector in the material. With enough such measurements, one can numerically

fit the sound velocities to the Christoffel equations and obtain all the independent stiffness tensor elements (and, in some cases, the refractive index). Measured values are fit, usually via a linear least square model, to solve for the individual coefficients. Some common stiffness tensor symmetries that have been used to describe biological samples are shown in Figure S2.

An analytic form of the Christoffel equations for a *cubic symmetry* can be obtained. When the wavevector, k, lies in the (001) plane at an angle ϕ relative to the x-axis these are given by:

$$V_L = \sqrt{\frac{\frac{1}{2}\{(c_{11}+c_{44}) \pm [(c_{11}-c_{44})^2 - 4A(c_{11}+c_{12})cos^2\phi\ sin^2\phi\ ]^{1/2}\}}{\rho}}$$

$$V_{T_2} = \sqrt{\frac{\frac{1}{2}\left\{(c_{11}+c_{44}) \pm [(c_{11}-c_{44})^2 - 4A(c_{11}+c_{12})cos^2\phi\ sin^2\phi\ ]^{\frac{1}{2}}\right\}}{\rho}}$$

$$V_{T_1} = \sqrt{\frac{c_{44}}{\rho}} \qquad \textbf{Eqn. S19}$$

Isotropic

$$\begin{pmatrix} c_{11} & c_{12} & c_{12} & 0 & 0 & 0 \\ c_{12} & c_{11} & c_{12} & 0 & 0 & 0 \\ c_{12} & c_{12} & c_{11} & 0 & 0 & 0 \\ 0 & 0 & 0 & \frac{1}{2}(c_{11}\text{-}c_{12}) & 0 & 0 \\ 0 & 0 & 0 & 0 & \frac{1}{2}(c_{11}\text{-}c_{12}) & 0 \\ 0 & 0 & 0 & 0 & 0 & \frac{1}{2}(c_{11}\text{-}c_{12}) \end{pmatrix}$$

Cubic

$$\begin{pmatrix} c_{11} & c_{12} & c_{12} & 0 & 0 & 0 \\ c_{12} & c_{11} & c_{12} & 0 & 0 & 0 \\ c_{12} & c_{12} & c_{11} & 0 & 0 & 0 \\ 0 & 0 & 0 & c_{44} & 0 & 0 \\ 0 & 0 & 0 & 0 & c_{44} & 0 \\ 0 & 0 & 0 & 0 & 0 & c_{44} \end{pmatrix}$$

Hexagonal

$$\begin{pmatrix} c_{11} & c_{12} & c_{13} & 0 & 0 & 0 \\ c_{12} & c_{11} & c_{13} & 0 & 0 & 0 \\ c_{13} & c_{13} & c_{11} & 0 & 0 & 0 \\ 0 & 0 & 0 & c_{44} & 0 & 0 \\ 0 & 0 & 0 & 0 & c_{44} & 0 \\ 0 & 0 & 0 & 0 & 0 & \frac{1}{2}(c_{11}\text{-}c_{12}) \end{pmatrix}$$

Figure S2. Three of the most common stiffness tensor symmetries used to describe biological systems measured with BLS. Note: In the isotropic, cubic and hexagonal cases there are only 2, 3 and 4 independent components respectively.

When on the other hand the wavevector, k, is in the (011) plane at an angle ϕ relative to the x-axis these will be given by:

$$V_L = \sqrt{\frac{\frac{1}{4}\{(c_{11}+c_{12}+4c_{44})+A_1cos^2\phi \pm [(c_{11}+c_{12})^2 - A_1(6c_{11}+14c_{12}+8c_{44})cos^2\phi + A_1(9c_{11}+15c_{12}+6c_{44})cos^4\phi\,]^{1/2}}{\rho}}$$

$$V_{T_1} = \sqrt{\frac{\frac{1}{4}\{(c_{11}+c_{12}+4c_{44})+A_1cos^2\phi \pm [(c_{11}+c_{12})^2 - A_1(6c_{11}+14c_{12}+8c_{44})cos^2\phi + A_1(9c_{11}+15c_{12}+6c_{44})cos^4\phi\,]^{1/2}}{\rho}}$$

$$V_{T_2} = \sqrt{\frac{\frac{1}{2}[(c_{11}-c_{12})-A_2cos^2\phi\,]}{\rho}} \qquad \textbf{Eqn. S20}$$

where $A_1 = c_{11} - c_{12} - 2c_{44}$ and $A_2 = c_{11} - c_{12} - 2c_{44}$. In each case one fits angle (ϕ) resolved measurements to extract the different unknown stiffness tensor coefficients. The bulk, Young's and shear elastic moduli can be calculated from:

$$K = \frac{(c_{11}+2c_{12})}{3} \qquad \textbf{Eqn. S21a}$$

$$E = \frac{(c_{11}-c_{12})(c_{11}+2c_{12})}{(c_{11}+c_{12})} \qquad \textbf{Eqn. S21b}$$

$$G = c_{44} = \frac{(c_{11}-c_{12})}{2} \qquad \textbf{Eqn. S21c}$$

The analytical form of the Christoffel equations for a system with *hexagonal symmetry* are given by:

$$V_L = \frac{c_{11}sin^2\phi + c_{33}cos^2\phi + c_{44} \pm \sqrt{[(c_{11}-c_{44})sin^2\phi + (c_{44} - c_{33})cos^2\phi\,]^2 + 4(c_{13}+c_{44})^2 sin^2\phi\, cos^2\phi}}{2\rho}$$

$$V_{T_1} = \frac{c_{11}\,sin^2\phi + c_{33}\,cos^2\phi + c_{44} \pm \sqrt{[(c_{11}-c_{44})sin^2\phi + (c_{44} - c_{33})\,cos^2\phi\,]^2 + 4(c_{13}+c_{44})^2 sin^2\phi\, cos^2\phi}}{2\rho}$$

$$V_{T_2} = \sqrt{\frac{c_{66}sin^2\phi + c_{44}cos^2\phi}{\rho}} \qquad \textbf{Eqn. S22}$$

Here the bulk, Young's and shear elastic moduli will be given by:

$$K = \frac{-2c_{13}^2 + (c_{11}+c_{12})c_{33}}{c_{11}+c_{12}-4c_{13}+2c_{33}} \qquad \textbf{Eqn. S23a}$$

$$E_{\parallel} = c_{33} - \frac{2c_{13}^2}{c_{11}+c_{12}} \qquad E_{\perp} = \frac{(c_{11}-c_{12})[c_{33}(c_{11}+c_{12})-2c_{13}^2]}{c_{11}c_{33}-c_{13}^2} \qquad \textbf{Eqn. S23b}$$

$$G_1 = c_{44} \qquad G_2 = c_{66}$$ **Eqn. S23c**

$$\sigma_{12} = \frac{c_{33}c_{12} - c_{13}^2}{c_{33}c_{11} - c_{13}^2} \qquad \sigma_{13} = \frac{c_{13}}{c_{11} + c_{12}}$$ **Eqn. S23d**

Registration to accepted/standard values

Different BLS instruments operate at different wavelengths and there are various extrinsic factors that can affect the BLS frequency shift (ν_B) and linewidth (Γ_B). It has previously been proposed that rather than simply reporting ν_B and Γ_B it may be desirable to report $\underline{\nu}_B = \nu_B/\nu_B^w - 1$ and $\underline{\Gamma}_B = \Gamma_B/\Gamma_B^w - 1$, where ν_B^w and Γ_B^w are the BLS frequency shift and linewidth of water measured using the same instrument, at the same temperatures and experimental conditions. The intention thereof was to allow for easy comparison of measurements from different instruments with different probing wavelengths, and to some degree also account for different artifacts that would perturb measurements of the probed sample in a similar way as water. These however do not account for constant systematic offsets or spectral broadening resulting from spectrometer design and measurement geometry. We thereby propose the following correction.

For ν_B a systematic offset can be accounted for by comparing the measured frequency shift for *pure* water (ideally from an injection grade ampoule, else double or triple distilled) $\nu_B^{(w)}$ measured with the same scattering geometry (with effective scattering wavevector $q^{(w)}$, Eqn. S5) as the sample, with an accepted value for water $\nu_B^{(w,0)}$ at the same temperature (T). We propose the following correction for the BLS frequency shift $\nu_B^{(a)}$ measured for sample "a" :

$$\nu'^{(a)}_B(T) = \nu_B^{(a)}(T) - \nu_B^{(w)}(T) + \left(q^{(w)}/q^{(w,0)}\right)\nu_B^{(w,0)}(T)$$ **Eqn. S24**

Where $\nu_B^{(w,0)}$is and accepted standard value of water, and $q^{(w,0)}$ the scattering wavevector of the standard value measurement. From this it follows that the corrected hypersonic speed $V'^{(a)}(T)$ will be given by:

$$V'^{(a)}(T) = (2\pi/q^{(a)})\left[\nu_B^{(a)}(T) - \nu_B^{(w)}(T) + \left(q^{(w)}/q^{(w,0)}\right)\nu_B^{(w,0)}(T)\right]$$ **Eqn. S25**

and the change in the hypersonic speed relative to the corrected value for water can compactly be written as:

$$\frac{V'^{(a)}(T)}{V'^{(w)}(T)} = \frac{q^{(w,0)}}{q^{(a)}}\left[\frac{\nu_B^{(a)}(T) - \nu_B^{(w)}(T)}{\nu_B^{(w,0)}(T)}\right] + \frac{q^{(w)}}{q^{(a)}}$$ **Eqn. S26**

An analogous correction can be applied to the linewidth:

$$\Gamma'^{(a)}_{B}(T) = \Gamma^{(a)}_{B}(T) - \Gamma^{(w)}_{B}(T) + \left(q^{(w)}/q^{(w,0)}\right)^2 \Gamma^{(w,0)}_{B}(T) \qquad \textbf{Eqn. S27}$$

Where $\Gamma^{(w,0)}_{B}$ is the accepted standard value of water. It follows that the corrected kinematic viscosity (μ_L) will be given by:

$$\mu'^{(a)}_{L}(T) = (2\pi/q^{(a)\,2})\left[\Gamma^{(a)}_{B}(T) - \Gamma^{(w)}_{B}(T) + \left(q^{(w)}/q^{(w,0)}\right)^2 \Gamma^{(w,0)}_{B}(T)\right] \qquad \textbf{Eqn. S28}$$

and the relative difference of the corrected kinematic viscosity to that of water will be:

$$\frac{\mu'^{(a)}_{L}(T)}{\mu'^{(w)}_{L}(T)} = \left(\frac{q^{(w,0)}}{q^{(a)}}\right)^2 \left[\frac{\Gamma^{(a)}_{B}(T) - \Gamma^{(w)}_{B}(T)}{\Gamma^{(w,0)}_{B}(T)}\right] + \left(\frac{q^{(w)}}{q^{(a)}}\right)^2 \qquad \textbf{Eqn. S29}$$

An interrelated table of *standard values* for $\nu^{(w,0)}_{B}(T)$, $\Gamma^{(w,0)}_{B}(T)$ and $q^{(w,0)}$ that may be used, are provided at DOI:10.6084/m9.figshare.27794913. These were obtained from deconvolved TFP measurements on injection grade water ampoules in laboratories in Vienna and Hannover. They were performed in ≤1°C temperature steps over the range 20-40°C, and throughout showed agreement of better than 0.1% with each other for both the frequency shift and linewidth. The acoustic speed derived from these values agreed with the accepted acoustic velocity in pure water to within 0.2% over the temperature range 25-35°C for which the latter was available.

Relation between refractive index and mass density

The *Clausius–Mossotti equation* or equivalently the *Lorenz-Lorentz equation*, relates the (macroscopic) optical properties of a material to its microscopic physical properties (molecular polarizability and molecular density), and has been shown to be applicable in diverse solid materials. In particular it predicts a relation between the refractive index (n) and the mass density (ρ):

$$\frac{(n^2-1)}{(n^2+2)} = \left(\frac{4\pi}{3}\right) N\alpha = \left(\frac{4\pi}{3}\right)\left(\frac{\rho}{M}\right)\alpha \qquad \textbf{Eqn. S30}$$

where N is the number density of molecules, M is their molecular weight, and α is the electronic polarizability. When the electronic polarizability or the number density of molecules is small (as $N\alpha \to 0$), this reduces to: $n^2 - 1 \approx 4\pi M^{-1}\alpha\rho$ and further to $n - 1 \approx 2\pi M^{-1}\alpha\rho$. These approximations have been found to be valid for gases under normal pressure. As can be seen none of these relations directly imply that n^2/ρ is constant (yet alone material independent) as would be desired for the BLS frequency shift to be related directly to the elastic modulus (Eqn. S10). As such independent measurements of both the refractive index

n and mass density ρ are in general required to obtain accurate prediction of viscoelastic moduli from BLS data.

Supplementary References

1 Berne, B. J. & Pecora, R. *Dynamic Light Scattering: With Applications to Chemistry, Biology, and Physics*. (Dover Publications, 2000).

2 Dil, J. G. Brillouin scattering in condensed matter. *Reports on Progress in Physics* **45**, 285-334 (1982). https://doi.org:10.1088/0034-4885/45/3/002

3 Koski, K. J. & Yarger, J. L. Brillouin imaging. *Applied Physics Letters* **87**, 061903 (2005). https://doi.org:10.1063/1.1999857

4 Scarcelli, G. & Yun, S. H. Confocal Brillouin microscopy for three-dimensional mechanical imaging. *Nat Photonics* **2**, 39-43 (2007). https://doi.org:10.1038/nphoton.2007.250

5 Massey, A. *et al.* Mechanical properties of human tumour tissues and their implications for cancer development. *Nature Reviews Physics* **6**, 269-282 (2024). https://doi.org:10.1038/s42254-024-00707-2

6 Tran, R. *et al.* Biomechanics of haemostasis and thrombosis in health and disease: from the macro- to molecular scale. *Journal of Cellular and Molecular Medicine* **17**, 579-596 (2013). https://doi.org:https://doi.org/10.1111/jcmm.12041

7 Antonacci, G. *et al.* Recent progress and current opinions in Brillouin microscopy for life science applications. *Biophysical Reviews* (2020). https://doi.org:10.1007/s12551-020-00701-9

8 Palombo, F. & Fioretto, D. Brillouin Light Scattering: Applications in Biomedical Sciences. *Chem Rev* **119**, 7833-7847 (2019). https://doi.org:10.1021/acs.chemrev.9b00019

9 Elsayad, K., Palombo, F., Dehoux, T. & Fioretto, D. Brillouin Light Scattering Microspectroscopy for Biomedical Research and Applications: introduction to feature issue. *Biomed Opt Express* **10**, 2670-2673 (2019). https://doi.org:10.1364/BOE.10.002670

10 Kabakova, I. *et al.* Brillouin microscopy. *Nature Reviews Methods Primers* **4**, 8 (2024). https://doi.org:10.1038/s43586-023-00286-z

11 Wu, P. J. *et al.* Water content, not stiffness, dominates Brillouin spectroscopy measurements in hydrated materials. *Nat Methods* **15**, 561-562 (2018). https://doi.org:10.1038/s41592-018-0076-1

12 Scarcelli, G. & Yun, S. H. Reply to 'Water content, not stiffness, dominates Brillouin spectroscopy measurements in hydrated materials'. *Nat Methods* **15**, 562-563 (2018). https://doi.org:10.1038/s41592-018-0075-2

13 Gutmann, M. *et al.* Beyond comparison: Brillouin microscopy and AFM-based indentation reveal divergent insights into the mechanical profile of the murine retina. *Journal of Physics: Photonics* **6**, 035020 (2024). https://doi.org:10.1088/2515-7647/ad5ae3

14 Yan, G., Monnier, S., Mouelhi, M. & Dehoux, T. Probing molecular crowding in compressed tissues with Brillouin light scattering. *Proceedings of the National Academy of Sciences* **119**, e2113614119 (2022). https://doi.org:doi:10.1073/pnas.2113614119

15 Keshmiri, H. *et al.* Brillouin light scattering anisotropy microscopy for imaging the viscoelastic anisotropy in living cells. *Nature Photonics* **18**, 276-285 (2024). https://doi.org:10.1038/s41566-023-01368-w

16 Eisenberger, P., Alexandropoulos, N. G. & Platzman, P. M. X-Ray Brillouin Scattering. *Physical Review Letters* **28**, 1519-1522 (1972). https://doi.org:10.1103/PhysRevLett.28.1519

17 Verkerk, P. Neutron brillouin scattering. *Neutron News* **1**, 21-21 (1990). https://doi.org:10.1080/10448639008210194

18 Koski, K. J., Akhenblit, P., McKiernan, K. & Yarger, J. L. Non-invasive determination of the complete elastic moduli of spider silks. *Nat Mater* **12**, 262-267 (2013). https://doi.org:10.1038/nmat3549

19 Palombo, F. *et al.* Biomechanics of fibrous proteins of the extracellular matrix studied by Brillouin scattering. *J R Soc Interface* **11**, 20140739 (2014). https://doi.org:10.1098/rsif.2014.0739
20 Czibula, C. *et al.* The elastic stiffness tensor of cellulosic viscose fibers measured with Brillouin spectroscopy. *Journal of Physics: Photonics* **6**, 035012 (2024). https://doi.org:10.1088/2515-7647/ad4cc6
21 Kim, M. *et al.* Shear Brillouin light scattering microscope. *Opt Express* **24**, 319-328 (2016). https://doi.org:10.1364/OE.24.000319
22 Boon, J. P. & Yip, S. *Molecular Hydrodynamics*. (Dover Publications, 1991).
23 Figgins, R. Inelastic light scattering in liquids: Brillouin scattering. *Contemporary Physics* **12**, 283-297 (1971). https://doi.org:10.1080/00107517108213717
24 Fiore, A., Bevilacqua, C. & Scarcelli, G. Direct Three-Dimensional Measurement of Refractive Index via Dual Photon-Phonon Scattering. *Phys Rev Lett* **122**, 103901 (2019). https://doi.org:10.1103/PhysRevLett.122.103901
25 Pochylski, M. Structural relaxation in the wave-vector dependence of the longitudinal rigidity modulus. *Biomed. Opt. Express* **10**, 1957-1964 (2019). https://doi.org:10.1364/BOE.10.001957
26 Remer, I., Shaashoua, R., Shemesh, N., Ben-Zvi, A. & Bilenca, A. High-sensitivity and high-specificity biomechanical imaging by stimulated Brillouin scattering microscopy. *Nat Methods* **17**, 913-916 (2020). https://doi.org:10.1038/s41592-020-0882-0
27 Park, Y., Depeursinge, C. & Popescu, G. Quantitative phase imaging in biomedicine. *Nature Photonics* **12**, 578-589 (2018). https://doi.org:10.1038/s41566-018-0253-x
28 Schürmann, M. *et al.* Refractive index measurements of single, spherical cells using digital holographic microscopy. *Methods Cell Biol* **125**, 143-159 (2015). https://doi.org:10.1016/bs.mcb.2014.10.016
29 Schürmann, M., Scholze, J., Müller, P., Guck, J. & Chan, C. J. Cell nuclei have lower refractive index and mass density than cytoplasm. *Journal of Biophotonics* **9**, 1068-1076 (2016). https://doi.org:10.1002/jbio.201500273
30 Oster, G. & Yamamoto, M. Density Gradient Techniques. *Chemical Reviews* **63**, 257-268 (1963). https://doi.org:10.1021/cr60223a003
31 Pádua, A. A. H., Fareleira, J. M. N. A., Calado, J. C. G. & Wakeham, W. A. Validation of an accurate vibrating-wire densimeter: Density and viscosity of liquids over wide ranges of temperature and pressure. *International Journal of Thermophysics* **17**, 781-802 (1996). https://doi.org:10.1007/BF01439190
32 Bailey, M. *et al.* Viscoelastic properties of biopolymer hydrogels determined by Brillouin spectroscopy: A probe of tissue micromechanics. *Science Advances* **6**, eabc1937 (2020). https://doi.org:10.1126/sciadv.abc1937
33 Vanderwal, J., Mudare, S. M. & Walton, D. Deconvolution of Brillouin spectra. *Optics Communications* **37**, 33-36 (1981). https://doi.org:https://doi.org/10.1016/0030-4018(81)90170-X
34 Huang, J. *et al.* Processing method of spectral measurement using F-P etalon and ICCD. *Opt. Express* **20**, 18568-18578 (2012). https://doi.org:10.1364/OE.20.018568
35 Meng, Z. & Yakovlev, V. V. Precise Determination of Brillouin Scattering Spectrum Using a Virtually Imaged Phase Array (VIPA) Spectrometer and Charge-Coupled Device (CCD) Camera. *Appl Spectrosc* **70**, 1356-1363 (2016). https://doi.org:10.1177/0003702816654050
36 Allan, D. W. Statistics of atomic frequency standards. *Proceedings of the IEEE* **54**, 221-230 (1966). https://doi.org:10.1109/PROC.1966.4634
37 Adib, G. A., Sabry, Y. M. & Khalil, D. Allan Variance Characterization of Compact Fourier Transform Infrared Spectrometers. *Appl Spectrosc* **77**, 734-743 (2023). https://doi.org:10.1177/00037028231174248
38 Coker, Z. *et al.* Assessing performance of modern Brillouin spectrometers. *Opt. Express* **26**, 2400-2409 (2018). https://doi.org:10.1364/OE.26.002400
39 Ballmann, C. W., Meng, Z. & Yakovlev, V. V. Nonlinear Brillouin spectroscopy: what makes it a better tool for biological viscoelastic measurements. *Biomed Opt Express* **10**, 1750-1759 (2019). https://doi.org:10.1364/BOE.10.001750

40 Mattarelli, M., Vassalli, M. & Caponi, S. Relevant Length Scales in Brillouin Imaging of Biomaterials: The Interplay between Phonons Propagation and Light Focalization. *ACS Photonics* **7**, 2319-2328 (2020). https://doi.org:10.1021/acsphotonics.0c00801
41 Passeri, A. A. *et al.* Size and environment: The effect of phonon localization on micro-Brillouin imaging. *Biomaterials Advances* **147**, 213341 (2023). https://doi.org:https://doi.org/10.1016/j.bioadv.2023.213341
42 Silvia Caponi, D. F., and Maurizio Mattarelli. On the actual spatial resolution of Brillouin Imaging. *Optics Letters* **(in press)** (2020).
43 Bevilacqua, C., Sanchez-Iranzo, H., Richter, D., Diz-Munoz, A. & Prevedel, R. Imaging mechanical properties of sub-micron ECM in live zebrafish using Brillouin microscopy. *Biomed Opt Express* **10**, 1420-1431 (2019). https://doi.org:10.1364/BOE.10.001420
44 McCreery, R. L. *Raman Spectroscopy for Chemical Analysis.* (Wiley, 2005).
45 Mattana, S. *et al.* Non-contact mechanical and chemical analysis of single living cells by microspectroscopic techniques. *Light: Science & Applications* **7**, 17139-17139 (2018). https://doi.org:10.1038/lsa.2017.139
46 Antonacci, G., Foreman, M. R., Paterson, C. & Török, P. Spectral broadening in Brillouin imaging. *Applied Physics Letters* **103**, 221105 (2013). https://doi.org:10.1063/1.4836477
47 Wäldchen, S., Lehmann, J., Klein, T., van de Linde, S. & Sauer, M. Light-induced cell damage in live-cell super-resolution microscopy. *Scientific Reports* **5**, 15348 (2015). https://doi.org:10.1038/srep15348
48 Shao, P. *et al.* Spatially-resolved Brillouin spectroscopy reveals biomechanical abnormalities in mild to advanced keratoconus in vivo. *Sci Rep* **9**, 7467 (2019). https://doi.org:10.1038/s41598-019-43811-5
49 Zhang, H., Asroui, L., Randleman, J. B. & Scarcelli, G. Motion-tracking Brillouin microscopy for in-vivo corneal biomechanics mapping. *Biomed. Opt. Express* **13**, 6196-6210 (2022). https://doi.org:10.1364/BOE.472053
50 Sussner, H. & Vacher, R. High-precision measurements of Brillouin scattering frequencies. *Applied Optics* **18**, 3815-3818 (1979). https://doi.org:10.1364/AO.18.003815
51 Caponi, S. *et al.* Electro-optic modulator for high resolution Brillouin scattering measurements. *Review of Scientific Instruments* **72**, 198-200 (2001). https://doi.org:10.1063/1.1329899
52 Pontecorvo, E. R., G; Zhang, L; Gala, F; Zanini, C.; Testi, C SISTEMA PERFEZIONATO DI RIVELAZIONE BRILLOUIN 102024000005674. Italy patent (2024).
53 Shijun, X., Weiner, A. M. & Lin, C. A dispersion law for virtually imaged phased-array spectral dispersers based on paraxial wave theory. *IEEE Journal of Quantum Electronics* **40**, 420-426 (2004). https://doi.org:10.1109/JQE.2004.825210
54 Cardinali MA, C. S., Mattarelli M, Loré S, Fioretto D. Brillouin micro spectroscopy and morpho mechanics of a hybrid lens. *SIF Congress 2023*, 330 (2023). https://doi.org:10.1393/ncc/i2024-24330-6
55 *"Evaluation of measurement data — Guide to the expression of uncertainty in measurement" by the Joint Committee for Guides in Metrology* <https://www.bipm.org/documents/20126/2071204/JCGM_100_2008_E.pdf/cb0ef43f-baa5-11cf-3f85-4dcd86f77bd6 > (2008).
56 Török, P. & Foreman, M. R. Precision and informational limits in inelastic optical spectroscopy. *Scientific Reports* **9**, 6140 (2019). https://doi.org:10.1038/s41598-019-42619-7
57 Cardinali, M. A., Caponi, S., Mattarelli, M. & Fioretto, D. Brillouin scattering from biomedical samples: the challenge of heterogeneity. *Journal of Physics: Photonics* **6**, 035009 (2024). https://doi.org:10.1088/2515-7647/ad4cc7
58 Alunni Cardinali, M. *et al.* Brillouin–Raman microspectroscopy for the morpho-mechanical imaging of human lamellar bone. *Journal of The Royal Society Interface* **19**, 20210642 (2022). https://doi.org:doi:10.1098/rsif.2021.0642
59 Still, T., Mattarelli, M., Kiefer, D., Fytas, G. & Montagna, M. Eigenvibrations of Submicrometer Colloidal Spheres. *The Journal of Physical Chemistry Letters* **1**, 2440-2444 (2010). https://doi.org:10.1021/jz100774b

60 Reed, B. W., Williams, D. R., Moser, B. P. & Koski, K. J. Chemically Tuning Quantized Acoustic Phonons in 2D Layered MoO3 Nanoribbons. *Nano Lett* **19**, 4406-4412 (2019). https://doi.org:10.1021/acs.nanolett.9b01068
61 Battistoni, A., Bencivenga, F., Fioretto, D. & Masciovecchio, C. Practical way to avoid spurious geometrical contributions in Brillouin light scattering experiments at variable scattering angles. *Optics Letters* **39**, 5858-5861 (2014). https://doi.org:10.1364/OL.39.005858
62 Mattarelli, M., Capponi, G., Passeri, A. A., Fioretto, D. & Caponi, S. Disentanglement of Multiple Scattering Contribution in Brillouin Microscopy. *ACS Photonics* **9**, 2087-2091 (2022). https://doi.org:10.1021/acsphotonics.2c00322
63 Hristov, J. Bio-Heat Models Revisited: Concepts, Derivations, Nondimensalization and Fractionalization Approaches. *Frontiers in Physics* **7** (2019). https://doi.org:10.3389/fphy.2019.00189
64 Pérez-Cota, F. *et al.* Picosecond ultrasonics for elasticity-based imaging and characterization of biological cells. *Journal of Applied Physics* **128** (2020). https://doi.org:10.1063/5.0023744
65 Antonacci, G. *et al.* Birefringence-induced phase delay enables Brillouin mechanical imaging in turbid media. *Nat Commun* **15**, 5202 (2024). https://doi.org:10.1038/s41467-024-49419-2
66 Vaughan, M. *The Fabry-Perot Interferometer: History, Theory, Practice and Applications*. 1st Edition edn, (Routledge, 1989).
67 Soltwisch, M., Sukmanowski, J. & Quitmann, D. Brillouin scattering on noncrystalline ZnCl2. *The Journal of Chemical Physics* **86**, 3207-3215 (1987). https://doi.org:10.1063/1.452031
68 Sandercock, J. R. Brillouin scattering study of SbSI using a double-passed, stabilised scanning interferometer. *Optics Communications* **2**, 73-76 (1970). https://doi.org:https://doi.org/10.1016/0030-4018(70)90047-7
69 Berghaus, K., Zhang, J., Yun, S. H. & Scarcelli, G. High-finesse sub-GHz-resolution spectrometer employing VIPA etalons of different dispersion. *Opt Lett* **40**, 4436-4439 (2015). https://doi.org:10.1364/OL.40.004436
70 Takagi, Y. & Gammon, R. W. Brillouin scattering in thin samples: Observation of backscattering components by 90° scattering. *Journal of Applied Physics* **61**, 2030-2034 (1987). https://doi.org:10.1063/1.338000
71 Zhang, Y., Reed, B. W., Chung, F. R. & Koski, K. J. Mesoscale elastic properties of marine sponge spicules. *J Struct Biol* **193**, 67-74 (2016). https://doi.org:10.1016/j.jsb.2015.11.009
72 Holmes, M. J., Parker, N. G. & Povey, M. J. W. Temperature dependence of bulk viscosity in water using acoustic spectroscopy. *Journal of Physics: Conference Series* **269**, 012011 (2011). https://doi.org:10.1088/1742-6596/269/1/012011
73 Scarponi, F. *et al.* High-Performance Versatile Setup for Simultaneous Brillouin-Raman Microspectroscopy. *Physical Review X* **7**, 031015 (2017). https://doi.org:10.1103/PhysRevX.7.031015
74 Elsayad, K. *et al.* Mapping the subcellular mechanical properties of live cells in tissues with fluorescence emission-Brillouin imaging. *Sci Signal* **9**, rs5 (2016). https://doi.org:10.1126/scisignal.aaf6326
75 Fales, A. M., Ilev, I. K. & Pfefer, T. J. Evaluation of standardized performance test methods for biomedical Raman spectroscopy. *J Biomed Opt* **27** (2021). https://doi.org:10.1117/1.Jbo.27.7.074705
76 Webb, J. N., Zhang, H., Sinha Roy, A., Randleman, J. B. & Scarcelli, G. Detecting Mechanical Anisotropy of the Cornea Using Brillouin Microscopy. *Transl Vis Sci Technol* **9**, 26 (2020). https://doi.org:10.1167/tvst.9.7.26
77 *HDF5 File format:*, <https://www.hdfgroup.org/solutions/hdf5/> (2024).
78 *Example HDF5 BLS-data files and generation codes* <www.biobrillouin.org/hdf5> (2024).
79 Yun, S. H. & Chernyak, D. Brillouin microscopy: assessing ocular tissue biomechanics. *Curr Opin Ophthalmol* **29**, 299-305 (2018). https://doi.org:10.1097/ICU.0000000000000489
80 Shao, P. *et al.* Spatially-resolved Brillouin spectroscopy reveals biomechanical abnormalities in mild to advanced keratoconus in vivo. *Scientific Reports* **9**, 7467 (2019). https://doi.org:10.1038/s41598-019-43811-5

81 Randleman, J. B. *et al.* Subclinical Keratoconus Detection and Characterization Using Motion-Tracking Brillouin Microscopy. *Ophthalmology* **131**, 310-321 (2024). https://doi.org:10.1016/j.ophtha.2023.10.011
82 Bevilacqua, C. *et al.* High-resolution line-scan Brillouin microscopy for live imaging of mechanical properties during embryo development. *Nature Methods* **20**, 755-760 (2023). https://doi.org:10.1038/s41592-023-01822-1
83 Zhang, J., Nikolic, M., Tanner, K. & Scarcelli, G. Rapid biomechanical imaging at low irradiation level via dual line-scanning Brillouin microscopy. *Nat Methods* **20**, 677-681 (2023). https://doi.org:10.1038/s41592-023-01816-z
84 Shaashoua, R. *et al.* Brillouin gain microscopy. *Nature Photonics* **18**, 836-841 (2024). https://doi.org:10.1038/s41566-024-01445-8
85 Krug, B., Koukourakis, N. & Czarske, J. W. Impulsive stimulated Brillouin microscopy for non-contact, fast mechanical investigations of hydrogels. *Opt. Express* **27**, 26910-26923 (2019). https://doi.org:10.1364/OE.27.026910
86 Fiore, A. & Scarcelli, G. Single etalon design for two-stage cross-axis VIPA spectroscopy. *Biomed Opt Express* **10**, 1475-1481 (2019). https://doi.org:10.1364/BOE.10.001475
87 Kabakova, I. V., Xiang, Y., Paterson, C. & Török, P. Fiber-integrated Brillouin microspectroscopy: Towards Brillouin endoscopy. *Journal of Innovative Optical Health Sciences* **10**, 1742002 (2017). https://doi.org:10.1142/s1793545817420020
88 Xiang, Y. *et al.* Background-free fibre optic Brillouin probe for remote mapping of micromechanics. *Biomed. Opt. Express* **11**, 6687-6687 (2020). https://doi.org:10.1364/boe.404535
89 Pruidze, P., Chayleva, E., Weninger, W. J. & Elsayad, K. Brillouin scattering spectroscopy for studying human anatomy: Towards in situ mechanical characterization of soft tissue. *J. Eur. Opt. Society-Rapid Publ.* **19**, 31 (2023).
90 Illibauer, J. *et al.* Diagnostic potential of blood plasma longitudinal viscosity measured using Brillouin light scattering. *Proceedings of the National Academy of Sciences* **121**, e2323016121 (2024). https://doi.org:doi:10.1073/pnas.2323016121
91 La Cavera, S. *et al.* Label-free Brillouin endo-microscopy for the quantitative 3D imaging of sub-micrometre biology. *Communications Biology* **7**, 451 (2024). https://doi.org:10.1038/s42003-024-06126-4
92 La Cavera, S., Pérez-Cota, F., Smith, R. J. & Clark, M. Phonon imaging in 3D with a fibre probe. *Light: Science & Applications* **10**, 91 (2021). https://doi.org:10.1038/s41377-021-00532-7
93 Ryu, S., Martino, N., Kwok, S. J. J., Bernstein, L. & Yun, S.-H. Label-free histological imaging of tissues using Brillouin light scattering contrast. *Biomed. Opt. Express* **12**, 1437-1448 (2021). https://doi.org:10.1364/BOE.414474
94 Martinez-Vidal, L. *et al.* Progressive alteration of murine bladder elasticity in actinic cystitis detected by Brillouin microscopy. *Scientific Reports* **14**, 484 (2024). https://doi.org:10.1038/s41598-023-51006-2
95 Mattana, S., Caponi, S., Tamagnini, F., Fioretto, D. & Palombo, F. Viscoelasticity of amyloid plaques in transgenic mouse brain studied by Brillouin microspectroscopy and correlative Raman analysis. *J Innov Opt Health Sci* **10** (2017). https://doi.org:10.1142/S1793545817420019
96 Fasciani, A. *et al.* MLL4-associated condensates counterbalance Polycomb-mediated nuclear mechanical stress in Kabuki syndrome. *Nature Genetics* **52**, 1397-1411 (2020). https://doi.org:10.1038/s41588-020-00724-8
97 Cikes, D. *et al.* PCYT2-regulated lipid biosynthesis is critical to muscle health and ageing. *Nat Metab* **5**, 495-515 (2023). https://doi.org:10.1038/s42255-023-00766-2
98 Steelman, Z., Meng, Z., Traverso, A. J. & Yakovlev, V. V. Brillouin spectroscopy as a new method of screening for increased CSF total protein during bacterial meningitis. *J Biophotonics* **8**, 408-414 (2015). https://doi.org:10.1002/jbio.201400047
99 Adichtchev, S. V. *et al.* Brillouin spectroscopy of biorelevant fluids in relation to viscosity and solute concentration. *Phys Rev E* **99**, 062410 (2019). https://doi.org:10.1103/PhysRevE.99.062410

100 Windberger, U., Sparer, A. & Elsayad, K. The role of plasma in the yield stress of blood. *Clin Hemorheol Microcirc* (2023). https://doi.org:10.3233/ch-231701
101 Weiner, A. M. Reply to Comment on “Generalized grating equation for virtually-imaged phased-array spectral dispersions&#x201D. *Applied Optics* **51**, 8187-8189 (2012). https://doi.org:10.1364/AO.51.008187
102 Scarcelli, G., Kim, P. & Yun, S. H. Cross-axis cascading of spectral dispersion. *Optics Letters* **33**, 2979-2981 (2008). https://doi.org:10.1364/OL.33.002979
103 Scarcelli, G. & Yun, S. H. Multistage VIPA etalons for high-extinction parallel Brillouin spectroscopy. *Opt Express* **19**, 10913-10922 (2011). https://doi.org:10.1364/OE.19.010913
104 Scarcelli, G. *et al.* Noncontact three-dimensional mapping of intracellular hydromechanical properties by Brillouin microscopy. *Nat Methods* **12**, 1132-1134 (2015). https://doi.org:10.1038/nmeth.3616
105 Edrei, E., Gather, M. C. & Scarcelli, G. Integration of spectral coronagraphy within VIPA-based spectrometers for high extinction Brillouin imaging. *Opt Express* **25**, 6895-6903 (2017). https://doi.org:10.1364/OE.25.006895
106 Antonacci, G., de Turris, V., Rosa, A. & Ruocco, G. Background-deflection Brillouin microscopy reveals altered biomechanics of intracellular stress granules by ALS protein FUS. *Commun Biol* **1**, 139 (2018). https://doi.org:10.1038/s42003-018-0148-x
107 Guerriero, G. *et al.* Predicting nanocarriers' efficacy in 3D models with Brillouin microscopy. *Nanoscale* **15**, 19255-19267 (2023). https://doi.org:10.1039/d3nr03502f
108 Shao, P., Besner, S., Zhang, J., Scarcelli, G. & Yun, S. H. Etalon filters for Brillouin microscopy of highly scattering tissues. *Opt Express* **24**, 22232-22238 (2016). https://doi.org:10.1364/OE.24.022232
109 Fiore, A., Zhang, J., Shao, P., Yun, S. H. & Scarcelli, G. High-extinction virtually imaged phased array-based Brillouin spectroscopy of turbid biological media. *Appl Phys Lett* **108**, 203701 (2016). https://doi.org:10.1063/1.4948353
110 Meng, Z., Traverso, A. J. & Yakovlev, V. V. Background clean-up in Brillouin microspectroscopy of scattering medium. *Opt Express* **22**, 5410-5415 (2014). https://doi.org:10.1364/OE.22.005410
111 Antonacci, G., Lepert, G., Paterson, C. & Török, P. Elastic suppression in Brillouin imaging by destructive interference. *Applied Physics Letters* **107**, 061102 (2015). https://doi.org:10.1063/1.4927400
112 Meek, A. T. *et al.* A multi-modal microscope for integrated mapping of cellular forces and Brillouin scattering with high resolution. *Journal of Physics: Photonics* **6**, 025012 (2024). https://doi.org:10.1088/2515-7647/ad3d1a
113 Taisuke, E., Yabuzaki, T., Kitano, M., Sato, T. & Ogawa, T. Frequency-locking of a CW dye laser to absorption lines of neon by a Faraday filter. *IEEE Journal of Quantum Electronics* **14**, 977-982 (1978). https://doi.org:10.1109/JQE.1978.1069734
114 Traverso, A. J. *et al.* Dual Raman-Brillouin Microscope for Chemical and Mechanical Characterization and Imaging. *Anal Chem* **87**, 7519-7523 (2015). https://doi.org:10.1021/acs.analchem.5b02104
115 Troyanova-Wood, M. A. & Yakovlev, V. V. Multi-wavelength excitation Brillouin spectroscopy. *IEEE J Sel Top Quantum Electron* **27** (2021). https://doi.org:10.1109/jstqe.2021.3071955
116 Lepert, G., Gouveia, R. M., Connon, C. J. & Paterson, C. Assessing corneal biomechanics with Brillouin spectro-microscopy. *Faraday Discussions* **187**, 415-428 (2016). https://doi.org:10.1039/C5FD00152H
117 Antonacci, G., Elsayad, K. & Polli, D. On-Chip Notch Filter on a Silicon Nitride Ring Resonator for Brillouin Spectroscopy. *ACS Photonics* **9**, 772-777 (2022). https://doi.org:10.1021/acsphotonics.2c00005
118 Bilenca, A., Prevedel, R. & Scarcelli, G. Current state of stimulated Brillouin scattering microscopy for the life sciences. *Journal of Physics: Photonics* **6**, 032001 (2024). https://doi.org:10.1088/2515-7647/ad5506
119 Ballmann, C. W. *et al.* Stimulated Brillouin Scattering Microscopic Imaging. *Sci Rep* **5**, 18139 (2015). https://doi.org:10.1038/srep18139

120 Chow, D. M. & Yun, S.-H. Pulsed stimulated Brillouin microscopy. *Opt. Express* **31**, 19818-19827 (2023). https://doi.org:10.1364/OE.489158
121 Yang, F. *et al.* Pulsed stimulated Brillouin microscopy enables high-sensitivity mechanical imaging of live and fragile biological specimens. *Nature Methods* **20**, 1971-1979 (2023). https://doi.org:10.1038/s41592-023-02054-z
122 Li, T., Li, F., Liu, X., Yakovlev, V. V. & Agarwal, G. S. Quantum-enhanced stimulated Brillouin scattering spectroscopy and imaging. *Optica* **9**, 959-964 (2022). https://doi.org:10.1364/OPTICA.467635
123 Remer, I. & Bilenca, A. Background-free Brillouin spectroscopy in scattering media at 780 nm via stimulated Brillouin scattering. *Opt Lett* **41**, 926-929 (2016). https://doi.org:10.1364/OL.41.000926
124 Ballmann, C. W., Meng, Z., Traverso, A. J., Scully, M. O. & Yakovlev, V. V. Impulsive Brillouin microscopy. *Optica* **4**, 124-128 (2017). https://doi.org:10.1364/OPTICA.4.000124
125 Meng, Z., Petrov, G. I. & Yakovlev, V. V. Flow cytometry using Brillouin imaging and sensing via time-resolved optical (BISTRO) measurements. *Analyst* **140**, 7160-7164 (2015). https://doi.org:10.1039/c5an01700a
126 Gusev, V. E. & Ruello, P. Advances in applications of time-domain Brillouin scattering for nanoscale imaging. *Applied Physics Reviews* **5** (2018). https://doi.org:10.1063/1.5017241
127 Pérez-Cota, F. *et al.* High resolution 3D imaging of living cells with sub-optical wavelength phonons. *Scientific Reports* **6**, 39326 (2016). https://doi.org:10.1038/srep39326
128 Pérez-Cota, F. *et al.* Thin-film optoacoustic transducers for subcellular Brillouin oscillation imaging of individual biological cells. *Applied Optics* **54**, 8388-8398 (2015). https://doi.org:10.1364/AO.54.008388
129 Tanaka, H. & Sonehara, T. Superheterodyne light beating spectroscopy for Rayleigh–Brillouin scattering using frequency-tunable lasers. *Review of Scientific Instruments* **73**, 1998-2010 (2002). https://doi.org:10.1063/1.1469671
130 Taylor, M. A., Kijas, A. W., Wang, Z., Lauko, J. & Rowan, A. E. Heterodyne Brillouin microscopy for biomechanical imaging. *Biomed. Opt. Express* **12**, 6259-6268 (2021). https://doi.org:10.1364/BOE.435869
